# Differential Mutation Incorporated Quantum Honey Badger Algorithm with Dynamic Opposite Learning and Laplace Crossover for Fuzzy Front-End Product Design

**DOI:** 10.3390/biomimetics9010021

**Published:** 2024-01-02

**Authors:** Jiaxu Huang, Haiqing Hu

**Affiliations:** School of Economics and Management, Xi’an University of Technology, Xi’an 710054, China; huhaiqing@xaut.edu.cn

**Keywords:** differential mutation operation, dynamic opposite learning strategy, dynamic Laplace crossover, honey badger algorithm, quantum local search

## Abstract

In this paper, a multi-strategy fusion enhanced Honey Badger algorithm (EHBA) is proposed to address the problem of easy convergence to local optima and difficulty in achieving fast convergence in the Honey Badger algorithm (HBA). The adoption of a dynamic opposite learning strategy broadens the search area of the population, enhances global search ability, and improves population diversity. In the honey harvesting stage of the honey badger (development), differential mutation strategies are combined, selectively introducing local quantum search strategies that enhance local search capabilities and improve population optimization accuracy, or introducing dynamic Laplacian crossover operators that can improve convergence speed, while reducing the odds of the HBA sinking into local optima. Through comparative experiments with other algorithms on the CEC2017, CEC2020, and CEC2022 test sets, and three engineering examples, EHBA has been verified to have good solving performance. From the comparative analysis of convergence graphs, box plots, and algorithm performance tests, it can be seen that compared with the other eight algorithms, EHBA has better results, significantly improving its optimization ability and convergence speed, and has good application prospects in the field of optimization problems.

## 1. Introduction

With the continuous innovation and development of technology in recent years, engineering problems in various fields such as social life and scientific research have generated many complex optimization solving needs [1,2,3,4], such as dynamic changes, nonlinearity, uncertainty, and high-dimensional. Traditional methods, including the gradient descent method, yoke gradient method, variational method, Newton’s method, and other methods, find it difficult to obtain optimal solutions to these problems within a certain time or accuracy, and are no longer able to meet practical needs. In addition, their efficiency is relatively low when solving real-world engineering problems with large search space and non-linearity. On the contrary, meta-heuristics (MHs) are stochastic optimization methods that do not require gradients. Due to their self-organizing, adaptive, and self-learning characteristics, they have demonstrated their ability to solve real-world engineering design problems in different fields. Therefore, with the continuous advancement of society and the development of artificial intelligence, optimization methods based on an MHs algorithm have been developed.

MHs methods solve optimization problems by simulating biological behavior, physical facts, and chemical phenomena. They are divided into four categories: Swarm Intelligence (SI) algorithms, Evolutionary Algorithms (EA) [5], Physics-based Algorithms (PhA) [6,7,8], and human-based algorithms [9,10,11]. Recently, these behaviors have been widely modeled in various optimization techniques, and their results are summarized in Table 1. Among them, SI is a kind of meta-heuristic algorithm that explores optimization by mimicking the swarm intelligence pattern of behavior of biological and non-living systems in nature [12]. It has advantages such as good parallelism, autonomous exploration, easy implementation, strong flexibility, and fewer parameters. In general, the structure of excellent SI optimization algorithms is simple. The simple theories and mathematical models originate from nature and solve practical problems by simulating nature. Additionally, it is easy to incorporate its variant methods in line with state-of-the-art algorithms. Second, these optimization algorithms can be considered as black boxes, which can solve optimization cases for a series of output values and given input values. Furthermore, an important characteristic of SI algorithms is their randomness, which means that they will find the entire variable space and effectively escape local optima. They seek the optimal result through probability search, without requiring too much prior knowledge or analyzing the internal laws and correlations of the data. It only needs to learn from the data itself, self-organize, and adaptively solve optimization problems, which are very suitable for solving NP complete problems [13].

In the last few years, SI was designated as a small branch of artificial intelligence, is widely used in areas like path planning, mechanical control, engineering scheduling, feature extraction, image processing, training MLP, etc. [14,15,16,17,18,19,20,21], and has achieved significant development. The No Free Lunch (NFL) theorem proposed by Wolpert et al. [22] logically proves that there is no algorithm that can solve all optimization problems. Therefore, research in the field of SI algorithms is very active, with many experts and scholars conducting research on improvements to current algorithms and new algorithms. Typical examples include Particle Swarm Optimization algorithms (PSO) [23] and Ant Colony Optimization (ACO) [24], which have been inferred from by the cooperative foraging behavior of bird and ant colonies, respectively. Over the past few years, a number of researchers have been involved in the development of SI, proposing various algorithms that simulate the habits of natural organisms. Yang et al. [25] presented the Bat Algorithm (BA) to simulate the bats’ behavior using sonar for detection and localization. Gandomi et al. [26] have developed the Cuckoo Search algorithm (CS) according to the reproductive characteristics of cuckoo birds; the reason why the optimal solution obtained by CS is much better than that obtained by existing methods is because CS uses unique search features. References [27,28,29] proposed the Grey Wolf Optimizer (GWO), Whale Optimization Algorithm (WOA), and Salp Swarm Algorithm (SSA) by simulating the hunting behavior of grey wolf, humpback whale, and salp, respectively. Compared with well-known meta-heuristic algorithms, the GWO algorithm can provide highly competitive results. The results of classical engineering design problems and practical applications have shown that this algorithm is suitable for challenging problems with unknown search spaces. Compared with existing meta-heuristic algorithms and traditional methods, WOA has strong competitiveness. The SSA can effectively improve the initial random solution and converge to the optimal solution. The results of actual case studies demonstrate the advantages of the proposed algorithm in solving real-world problems with difficult and unknown search spaces.

Mirjalili et al. [30] proposed the Sea-horse Optimizer (SHO) from the motor, predatory, and reproductive behavior of the sea-horse. These three intelligent behaviors are expressed and constructed mathematically to balance the local development and global exploration of SHO. The experimental results indicate that SHO is a high-performance optimizer with positive adaptability for handling constraint problems. Abualigaha et al. [31] introduced the Reptile Search Algorithm (RSA) derived from the hunting activity of crocodiles, the search method of RSA is unique, and it achieves better results. Based on mathematical models of sine and cosine functions, the Sine Cosine Algorithm (SCA) [32] is proposed, which can effectively explore different regions of the search space, avoid local optima, converge to global optima, and effectively utilize the promising regions of the search space during the optimization process. The SCA algorithm has obtained smooth shapes of airfoils with very low drag, indicating its effectiveness in solving practical problems with constraints and unknown search spaces. Subsequently, Tunicate Swarm Algorithm (TSA) [33], Wild Horse Optimizer [34] (WHO), Archimedes Optimization Algorithm (AOA) [35], and Moth Flame Optimization (MFO) [36] were successively proposed.

**Table 1:** A brief review of meta-heuristic algorithms.

Algorithms	Abbrev.	Inspiration
Particle Swarm Optimization	PSO [23]	The predation behavior of birds
Genetic algorithms	GA [5]	Darwin’s theory
Gravitational Search Algorithm	GSA [6]	The interaction law
Teaching Learning-Based Optimization	TLBO [8]	The effect of influence of a teacher on learners
Ant Colony Optimization	ACO [24]	The foraging behavior of ants
Bat Algorithm	BA [25]	The echolocation behavior of bats
Cuckoo Search algorithm	CS [26]	The reproductive characteristics of cuckoo birds
Gray Wolf Optimization	GWO [27]	The leadership hierarchy and hunting mechanism
Whale Optimization Algorithm	WOA [28]	The bubble-net hunting behavior of humpback whales
Salp Swarm Algorithm	SSA [29]	The swarming behaviour of salps when navigating and foraging in oceans
Sea-horse Optimizer	SHO [30]	The movement, predation, and breeding behaviors of sea horses
Reptile Search Algorithm	RSA [31]	The hunting behavior of crocodiles
Tunicate Swarm Algorithm	TSA [33]	The group behavior of tunicates in the ocean
Sine Cosine Algorithm	SCA [32]	Based on mathematical models of sine and cosine functions
Wild Horse Optimizer	WHO [34]	The decency behaviour of the horse
Arithmetic Optimization Algorithm	AOA [35]	The main arithmetic operators in mathematics
Moth Flame Optimization	MFO [36]	The navigation method of moths
Honey Badger Algorithm	HBA [37]	The intelligent foraging behavior of honey badger

The basic framework of the MHs algorithm mentioned above is established in two stages, namely the exploration and exploration stages. The MHs algorithm needs to achieve a perfect balance between these stages in order to be efficient and robust, thereby ensuring the best results in one or more specific applications. The exploration process involves searching for regions of distant feasible solutions to ensure obtaining better candidate solutions. After the exploration phase, exploring the search space is crucial. This algorithm will converge to a promising solution and is expected to find the best solution through local convergence strategy [19].

A good balance of exploration and exploitation and prevention of falling into local solutions are the key requirements for MHs algorithms to solve engineering optimization problems. They ensure a large search space and the acquisition of the optimal global solution. The summary results show that researchers mainly deal with (1) mixing two or more other strategies. The improved meta-heuristic algorithm will introduce the advantages and disadvantages of each algorithm, and refer to the corresponding strategies in a targeted manner to improve optimization efficiency. (2) Propose new heuristic optimization algorithms that are more adaptable to complex engineering optimization problems. However, the newly proposed algorithm must be more mature and generalizable for optimization problems when migrating to a new project.

The Honey Badger Algorithm (HBA) [37] was developed by Fatma et al. Firstly, the special feature of HBA from other meta-heuristic algorithms lies in the use of two new mechanisms to update individual positions: the foraging behavior of honey badgers in both mining and honey picking modes, which possesses stronger searching ability and performs well for the complex practical problems. The dynamic search behavior of honey badger with digging and honey finding approaches are formulated into exploration and exploitation phases in HBA.

Secondly, compared with different algorithms such as PSO, WOA, and GWO, etc., HBA has been widely noticed and used in various fields because of its high flexibility, simple algorithm structure, high convergence accuracy, and operability. It has a stronger searching ability and performs well for the complex practical problems. HBA has successfully solved the speed reducer design problem, tension/compression spring design problem, and some other constraint engineering problems.

Therefore, experts have made varying degrees of improvements to it in order to better adapt to various problems in recent times. For example, Akdag et al. proposed a developed honey badger algorithm (DHBA) to solve the optimal power flow problem [38]. Han et al. proposed an improved chaotic honey badger algorithm to optimize and efficiently model proton exchange membrane fuel cells [39]. The literature [40] proposes an enhanced HBA (LHBA) based on Levy flight strategy and applies it to the optimization problem of robot grippers, The results show that LHBA can obtain the minimum value of the difference between the minimum force and the maximum force and successfully solve this optimization problem. In order to improve the overall optimization performance of basic HBA, literature [41] proposes an improved HBA named SaCHBA_PDN based on the Bernoulli shift graph, segmented optimal decreasing neighborhood, and policy adaptive horizontal crossing and applies it to solve the path planning problem of unmanned aerial vehicles (UAVs). Test experimental results show that SaCHBA_PDN has a better performance than other optimization algorithms. Simulation results show that SaCHBA_PDN can obtain more feasible and efficient paths in different obstacle environments, etc. However, the HBA still has limitations in falling into local optima and solving accuracy when facing multiple local solution problems [37], while the experimental results in this paper also show that there is some room for improvement in its performance such as optimization accuracy and stability. Therefore, this paper attempts to improve some limitations of the HBA.

To further improve the performance of the original HBA, it was enhanced by combining four different strategies: dynamic opposite learning, differential mutation operations, local quantum search, and dynamic Laplacian crossover operators, forming the enhanced Honey Badger Algorithm (EHBA) to be studied in this paper. What is more, the EHBA has been successfully introduced to a number of typical practical engineering problems. To summarize, the main contributions made include:

(a) A dynamic opposite learning strategy was adopted for HBA initialization to enhance the diversity of the population and quality of candidate solution for performance improvement of the original HBA, and increases the convergence speed of the algorithm.
(b) Combining differential mutation operations to increase the diversity of individual populations, enhance the HBA’s capability to jump out of local optima, and to some extent increase the precision of HBA.
(c) Local quantum search and dynamic Laplacian crossover operators are selectively used in the mining and honey mining stages to balance the development and exploration stages of the algorithm.
(d) Performance testing and analysis of EHBA were conducted on test sets CEC2017, CEC2020, and CEC2022, respectively. The feasibility, stability, and high accuracy of the proposed method have been verified through existing test sets. Improved new algorithms EHBA were adopted to design and solve three typical engineering practical cases, further verifying the practicality of EHBA.

The rest of the research content of this article is as outlined below: Section 2 outlines the basic theory of the original honey badger algorithm, combining multiple strategies to establish an enhanced honey badger algorithm (EHBA), and provides specific process steps for improving the algorithm in Section 3; for the effectiveness of the developed EHBA, calculated and statistical analyses were conducted in Section 4 using test sets CEC2017, CEC2020, and CEC2022, respectively; Section 5 provides three specific engineering examples and analysis to validate the engineering utility of EHBA; Finally, the conclusion and future research of the entire article was made in Section 6.

## 2. Theoretical Basis of Honey Badger Algorithm

The HBA simulates the foraging behavior of honey badgers in both digging and honey modes. In the previous mode, honey badger employs its olfactory capabilities to approach the prey’s position. As it approaches, the honey badger moves around prey to select suitable places to excavate and capture it. With the second option, the honey badger directly tracks the honeycomb under the guidance of the honey guide bird. In theory, HBA has both exploration and exploitation stages, so it can be called a global optimization algorithm. The feeding activity of the honey badger exhibits the properties of powerful optimization capacity and rapid convergence rate.

### 2.1. Population Initialization Stage

As with all meta-heuristics, HBA starts the optimization process by generating a uniformly distributed randomized population within a set boundary range. According to Equation (1), initialize the population and individual position of honey badgers.

(1) $$P_{i}=r_{1}\times (Upb_{i}-Lob_{i})+Lob_{i},$$

where *r*_1_ is a random value within [0, 1], $Lob_{i}$ and $Upb_{i}$ represent the lower and upper bound of the problem to be solved, respectively.

Forming the initial population matrix ***P*** in below Equation (2).

(2) $$P=\left[\begin{array}{c} P_{1}\\ P_{2}\\ \cdots \\ \begin{array}{l} \vdots \\ \cdots \\ P_{N} \end{array} \end{array}\right]=\left[\begin{array}{ccccc} p_{1,1} & \cdots & p_{1,j} & p_{1,Dim-1} & p_{1,Dim}\\ p_{2,1} & \cdots & p_{2,j} & \cdots & p_{2,Dim}\\ \cdots & \cdots & p_{i,j} & \cdots & \cdots \\ \vdots & \vdots & \vdots & \vdots & \vdots \\ p_{N-1,1} & \cdots & p_{N-1,j} & \cdots & p_{N-1,Dim}\\ p_{N,1} & \cdots & p_{N,j} & p_{N,Dim-1} & p_{N,Dim} \end{array}\right]\;,$$

where ***P****_i_* (*i* = 1, 2, …, *N*) represents the candidate solution position vector, and ***P****_i,j_* represents the position in the *j*-th direction of the *i*-th candidate honey badger.

As mentioned earlier, there are two parts to update the position process in the HBA, namely the “digging stage” and the “honey stage”.

### 2.2. Digging Stage (Exploration)

Some honey badgers approach their prey through their sense of smell, and this unique foraging behavior provides us with the direction of the digging stage. In addition to the location update formula, the digging stage also defines three related concepts: intensity operator, trend modifier, and density factor. 

#### 2.2.1. Definition of the Intensity ***I***

The intensity ***I*** is proportional to the density of prey and the distance between it and the honey badger, and is denoted by the inverse square law [42] in Equation (4).

(3) $$S={\left(P_{i}-P_{i+1}\right)}^{2},$$

(4) $$I_{i}=r_{2}\frac{S}{4\pi d_{i}^{2}},$$

(5) $$d_{i}=P_{prey}-P_{i},$$

where ***I**_i_* is the intensity of the prey’s odor in Equation (4). If the odor is strong, the speed of the movement will be rapid, and vice versa. *S* and ***d****_i_* indicate the source or concentration intensity (location of prey) and the distance between the current honey badger candidate and the prey, *r*_2_, is a random number between 0 and 1.

#### 2.2.2. Update Density Factor *α*

The density factor (*α*) governs the time-varying stochasticity to guarantee a steady shift from exploration to exploitation. The diminishing factor α is refreshed to lower the stochasticity over time using Equation (6), which reduces with iterations.

(6) $$\alpha =C\times \exp(\frac{-t}{T}),$$

where *T* and *t* are the maximum number of iterations and the current number of iterations, respectively. *C* default is 2.

#### 2.2.3. Definition of the Search Orientation ***F***

The next few steps are all used to jump out of the local optimum zone. Here, the EHBA uses a flag ***F*** that switches the search orientation to take advantage of numerous opportunities for individuals to strictly scan the search space.

(7) $$F=\left\{ \begin{array}{l} 1,\;\mathrm{if}\;r_{3}\leq 0.5\\ -1,\;\mathrm{otherwise} \end{array}\right.,$$

where *r*_3_ is random numbers in the range of 0–1.

#### 2.2.4. Update Location of Digging Stage

During the digging period, honey badgers depend strongly on the scent intensity, the distance between them, and search factors *α*. Badgers may be subject to any interference during excavation activities; this can be a hindrance to their search for better prey sites. The honey badger executes actions that resemble the shape of a heart-line. Equation (8) can be used to mimic cardioid movement.

(8) $$P_{New}=P_{prey}+F\times \beta \times P_{prey}+F\times r_{3}\times \alpha \times d_{i}\times \left|\cos(2\pi r_{4})\times [1-\cos(2\pi r_{5})]\right|,$$

among them, ***P****_prey_* is the globally optimal prey location so far. *β* ≥ 1 (default to 6) is the honey badger’s capacity to forage, ***d****_i_* is shown in Equation (5). *r*_4_, *r*_5,_ and *r*_6_ are three different random numbers between 0 and 1, respectively. ***F*** is defined as a sign to change the search direction.

### 2.3. Honey Harvesting Stage (Exploitation)

The situation where the honey badger follows the honey guide badger to the hive is illustrated in Equation (9).

(9) $$P_{New}=P_{prey}+F\times r_{7}\times \alpha \times d_{i},$$

where ***P****_New_* and ***P****_prey_* represent the current individual location and the location of the prey, and *r*_7_ is random numbers in the range of 0–1. ***F*** and *α* are defined by Equations (8) and (6), respectively. From Equation (9), it can be observed that the honey badger near ***P****_prey_* is based on variable ***d****_i_*.

## 3. An Enhanced Honey Badger Algorithm Combining Multiple Strategies

To address the issue of inadequate precision in solving the original HBA, there is a phenomenon of insufficient global exploration capability and difficulty in jumping out of local extremes. An enhanced honey badger search algorithm is proposed by combining dynamic opposite learning strategy, differential mutation strategy, Laplacian, and quantum local strategy. Firstly, in terms of population initialization, dynamic opposite learning strategies are utilized to enhance the richness of the initial population. The differential mutation operation is to increase the diversity of the population and prevent HBA from falling into local optima. Simultaneously, introducing quantum local search or Laplacian operators for dynamic crossover operations in the local development stage allows the optimal honey badger individual to adopt different crossover strategies at different stages of development, with a fine search range in the early stage and better jumping out of local extremum in the later stage.

### 3.1. Dynamic Opposite Learning Strategy

Opposite learning is considered a new technology in intelligent computing, aims to find corresponding opposite solutions based on the current solution, and then select and save better solutions through fitness calculation. Initializing through opposite learning strategies can effectively improve the diversity of the population and help it escape from local optima.

In meta-heuristic optimization algorithms, the population initialization is usually randomly produced, which can only ensure the distribution of the population, but cannot assure the quality of the initial solution. Nevertheless, studies have shown that the quality of the initialization significantly affects the convergence rate and precision of HBA. Based on this issue, domestic and foreign scholars have introduced various strategies into the initialization part to enhance the initial performance, commonly including chaotic initialization, opposite learning, and Cauchy random generation, etc. This section introduces a dynamic opposite learning strategy to stronger the quality of initialization solutions [43] with Equation (10).

(10) $$P_{Dobl}=P_{Init}\times r_{8}\times (r_{9}\times (Upb+Lob-P_{Init}))-P_{Init},$$

where ***P****_Init_* and ***P****_Dobl_* are the population created at random and opposite initial population. *r*_8_ and *r*_9_ are the different arbitrary number within (0, 1). Firstly, ***P****_Init_* and ***P****_Dobl_* are produced, respectively. Then, merge them into a new population ***P****_New_* = {***P****_Dobl_* ∪ ***P****_Init_*}. Calculate the objective function of ***P****_New_*, and use a greedy strategy to fully compete within the population, selecting the best *N* candidate honey badgers as the initial population. This allows the population to near the optimal solution more quickly, thereby accelerating the convergence of the HBA.

### 3.2. Differential Mutation Operation

Differential evolution algorithm (DE) is a new parallel evolutionary algorithm. It consists of three operations: mutation, crossover, and selection [44,45,46]. The DE algorithm keeps the best individuals and eliminates the worst individuals by means of successive iterative operations, and leads the search process towards the global optimal solution. The concrete procedures of the three operations are described below:

- Mutations. Mutation refers to calculating the weighted position difference between two individuals in a population, then adding the position of a random individual to generate a mutated individual. The specific mutation procedures can be described with Equation (11).

(11) $$v_{i}^{t+1}=p_{{\bar{r}}_{1}}^{t}+F_{s}\times (p_{{\bar{r}}_{2}}^{t}-p_{{\bar{r}}_{2}}^{t}),$$

where $p_{{\bar{r}}_{1}}^{t},p_{{\bar{r}}_{2}}^{t},p_{{\bar{r}}_{3}}^{t}$ are individuals that are different from each other in the *t*-th iteration, respectively, ***F****_s_* represents the adaptive adjustment mutation operator.

- Crossover. By using some parts of the present population and corresponding parts of the mutant population, and exchanging them in accordance with certain rules, it is possible to make a cross population $m_{i}^{t}$ that can enrich the variety of the species in the population.

(12) $$m_{i}^{t+1}=\left\{ \begin{array}{l} v_{i}^{t+1}\;\mathrm{if}\;r_{j}<CR\;\mathrm{or}\;j=j_{rand}\\ p_{i}^{t}\;\mathrm{otherwise} \end{array}\right.,$$

where *CR* ∈ [0, 1] is the crossover probability, $r_{j}$ and $j_{rand}$ are random integer numbers in the range [0, 1] and [1,Dim] , respectively.

- Selection. If the fitness value of the cross vector $m_{i}^{t}$ is not inferior to the fitness value of the target individuals $p_{i}^{t}$ , then replace the target individual with the cross vector in the next generation.

(13) $$p_{i}^{t+1}=\left\{ \begin{array}{l} m_{i}^{t}\;\mathrm{if}\;f(m_{i}^{t})<f(p_{i}^{t})\\ p_{i}^{t}\;\mathrm{otherwise} \end{array}\right.,$$

The DE algorithm uses the variation information between individuals to disturb them, thereby increasing the variety of the individuals and searching for the optimum result. It has the merits of simplicity of processing, stability of search, and ease of implementation. In this contribution, the new individual obtained by the DE algorithm is substituted for the optimal individual in the original HBA and then drives the evolution process of the individuals. This not only enhances the precision and exploration, but also secures the convergence rate of the HBA.

### 3.3. Quantum Local Search

First, calculate the adaptive expansion coefficient of the current generation [47]:

(14) $$\beta (t)=(\beta_{\max}-\beta_{\min})\frac{T-t}{T}+\beta_{\min},$$

where *β*_max_ = 1 and *β*_min_ = 0.5 are the maximum and minimum values of the preset adaptive expansion coefficient. Generate an attraction point ***Q****_i_* based on individual historical average optimal position and group historical optimal position:

(15) $$Q_{i}=\varphi \cdot \bar{P}+(1-\varphi )P_{b}.$$

Assuming that the position vector of each honey badger has quantum behavior, the state of the position vector is described using the wave function ***φ***(*x*, *t*). The position equation of the new position vector obtained through Monte Carlo random simulation can be seen in Equation (16).

(16) $$P(t+1)=Q_{i}\pm \beta \left|\bar{P}-P\right|\cdot \ln(1/u).$$

In Equations (15) and (16), ***φ*** and ***u*** represents a *D* random number matrix that follows a uniform distribution from 0 to 1. The introduced quantum local search strategy generates an attraction point according to the Equation (15), and the honey badger population moves in a one-dimensional potential well centered around this attraction point, expanding the variety of new individuals and making sure the individuals of the honey badger population have better distribution. Finally, the position is updated according to Equation (16) to decrease the possibility of HBA entering local optima, making the HBA have better exploration performance, which is beneficial for balancing exploration and development. 

### 3.4. Dynamic Laplace Crossover

The Laplace crossover operator was proposed by Kusum et al. [48,49]. The density function of the Laplace operator distribution can be described in Equation (17).

(17) $$D(p)=\frac{1}{2b}\exp(-\frac{\left|p-a\right|}{b}).$$

In Equation (17), *a*∈*R* is the positional parameter, usually taken as 0, and *b* > 0 is the proportional parameter. First, generate a random number equally distributed on the interval [0, 1], and *λ* (random number) is generated by the Equation (18).

(18) $$\lambda =\left\{ \begin{array}{l} a-b\log_{e}(\varepsilon ),\varepsilon \leq \frac{1}{2}\\ a+b\log_{e}(\varepsilon ),\varepsilon >\frac{1}{2} \end{array}\right.,$$

In Laplacian crossover, two offspring $o^{\left(1\right)}=o_{1}^{\left(1\right)},o_{2}^{\left(1\right)},\cdots ,o_{n}^{\left(1\right)}$ and $o^{\left(2\right)}=o_{1}^{\left(2\right)},o_{2}^{\left(2\right)},\cdots ,o_{n}^{\left(2\right)}$ are generated by a pair of parents $p^{\left(2\right)}=p_{1}^{\left(2\right)},p_{2}^{\left(2\right)},\cdots ,p_{n}^{\left(2\right)}$ and $p^{\left(1\right)}=p_{1}^{\left(1\right)},p_{2}^{\left(1\right)},\cdots ,p_{n}^{\left(1\right)}$ by Equations (19) and (20).

(19) $$p_{i}^{\left(1\right)}=o_{i}^{\left(1\right)}+\lambda \left|o_{i}^{\left(1\right)}-o_{i}^{\left(1\right)}\right|,$$

(20) $$p_{i}^{\left(2\right)}=o_{i}^{\left(2\right)}+\lambda \left|o_{i}^{\left(2\right)}-o_{i}^{\left(2\right)}\right|.$$

**Figure 1:**
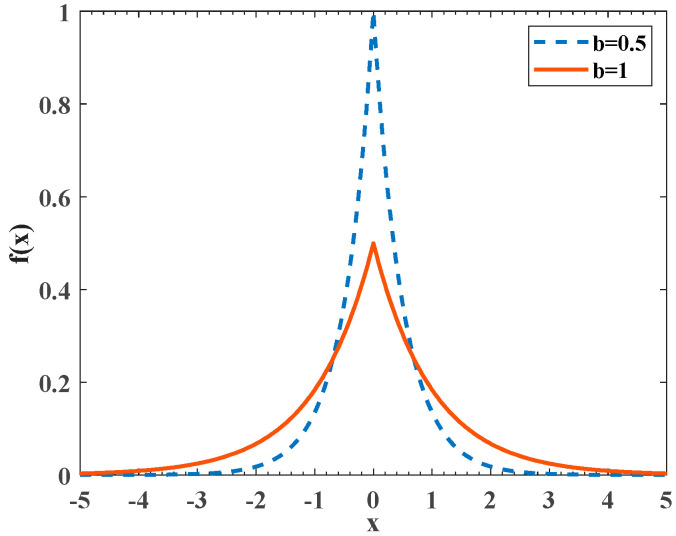
Laplace density function curve.

In order to match the iterative law of the algorithm, this article adopts a dynamic Laplacian crossover strategy for cross mutation operations. Figure 1 shows the differences in the Laplace cross density function curves under different values of *b*. In which, the solid line and dashed line are represented as *b* = 1 and *b* = 0.5. From a vertical perspective, the peak near the center value of the dashed line is greater than the solid line, and the peak at both ends is smaller than the solid line; from a horizontal perspective, the solid line descends more slowly as it approaches both ends of the horizontal axis, while the dashed line approaches both ends of the horizontal axis.

To make the algorithm easy to operate and universal, this article dynamically introduces the Laplace crossover operator in the local discovery stage to help the honey badger population break free from the constraints of local extremum and avoid premature convergence. Because *b* = 1 is more likely to generate random numbers entering and leaving the origin than *b* = 0.5, and has a wider distribution range, selecting *b* = 1 for Laplace crossing in the early stage of local exploration allows honey badger individuals to search the range of solutions with a larger step size and better break free from the constraints of local extremum by Equation (21).

(21) $$\lambda =\left\{ \begin{array}{l} a-\log_{e}(\varepsilon ),\varepsilon \leq \frac{1}{2}\\ a+\log_{e}(\varepsilon ),\varepsilon >\frac{1}{2} \end{array}\right.,\;r\leq 1-\frac{t}{T}.$$

Due to the high probability of generating random numbers near the central value of *b* = 0.5, in the later stage of local development, *b* = 0.5 is chosen for Laplacian crossover, which allows honey badger individuals to walk around the optimal solution with a smaller step length, fine search region, and improve the probability of finding the global optimal. The specific expression is defined with Equation (22).

(22) $$\lambda =\left\{ \begin{array}{l} a-\frac{1}{2}\log_{e}(\varepsilon ),\varepsilon \leq \frac{1}{2}\\ a+\frac{1}{2}\log_{e}(\varepsilon ),\varepsilon >\frac{1}{2} \end{array}\right.,\;r>1-\frac{t}{T}.$$

In Equations (21) and (22), 1 − *t*/*T* represents a monotonically decreasing function between [0, 1]. In the early stages of development, *t* is small, so 1 − *t*/*T* is large. The algorithm randomly selects a honey badger and the current optimal individual to perform a crossover operation according to Equation (21) when 1 − *t*/*T* is greater than *r*. In the later stage of development, *t* is large, so 1 − *t*/*T* is small. The algorithm randomly selects a honey badger and performs a crossover operation with the current optimal individual according to Equation (22) when it is less than *r*. The dynamic Laplacian crossover operation allows for the generation of offspring in the early stage, which can better explore the search space with a larger step size, increase the probability of jumping out of the local extremum, and avoid premature convergence. In the later stage of development, offspring that are closer to their parents are generated, which can finely search the space near the optimal solution with a smaller step size, increasing the probability of finding the global optimal solution, and helping the honey badger individuality converge to the global optimal at a faster speed. Overall, the introduction of Laplace crossover operator in the development stage enables the honey badger population to perform adaptive dynamic crossover operations according to the iterative process, improving the convergence rate and solving ability.

### 3.5. The Specific Steps of the Enhanced Honey Badger Algorithm

Initialize the population using dynamic opposite learning, replace the random method of HBA, and reduce the uncertainty of the algorithm. This initialization population strategy can generate high-quality populations with good diversity, laying the foundation for subsequent iterations. Introducing Laplacian crossover strategy or local quantum search strategy in the local exploration stage forces the honey badger group to adaptively select and update strategies during the iteration process, helping the honey badger group converge to the global optimum faster and decreasing the probability of premature convergence of the HBA. 

Step One. Initialize the population by Equation (1), perform the dynamic opposite learning population with Equation (10), and retain the optimal individuals according to the greedy strategy to enter the main program iteration.

Step Two. Calculate the objective function value of each honey badger, and record the optimal objective function value ***F****_Best_* and the optimal individual position ***X****_Best_* based on the results.

Step Three. Define intensity *I* with Equation (4) and density factor by Equation (6).

Step Four. Perform differential mutation operation Equations (11)–(13). 

Step Five. If r<0.5 , update the values through the digging stage with Equation (8).

Step six. If the random number r1>1−t/T , update the individual position of the population based on the quantum local search Equation (16); otherwise, if the random number $r_{2}>1-t/T$ , replace the individual position based on the dynamic Laplace crossover Equation (22); if r2≤1−t/T , update the individual position following the Equation (21).

Step Seven. After updating, judge whether it exceeds the upper and lower bounds of the position. If a certain dimension of the individual exceeds the upper bound, replace its value with the upper bound ***Upb***. If a certain dimension of the individual exceeds the lower bound, replace its value with the lower bound ***Lob***.

Step Eight. Evaluate the fitness value and judge whether is better than ***F****_Best_*; if it is better than ***F****_Best_*, the fitness value of this candidate solution is recorded as a new ***F****_Best_*, and the individual position is updated as ***P****_Best_*;

Step Nine. As the number of iteration increases, if t<T , return to Step Three; otherwise, output the optimal value ***F****_Best_* and the corresponding position ***P****_Best_*.

**Figure 2:**
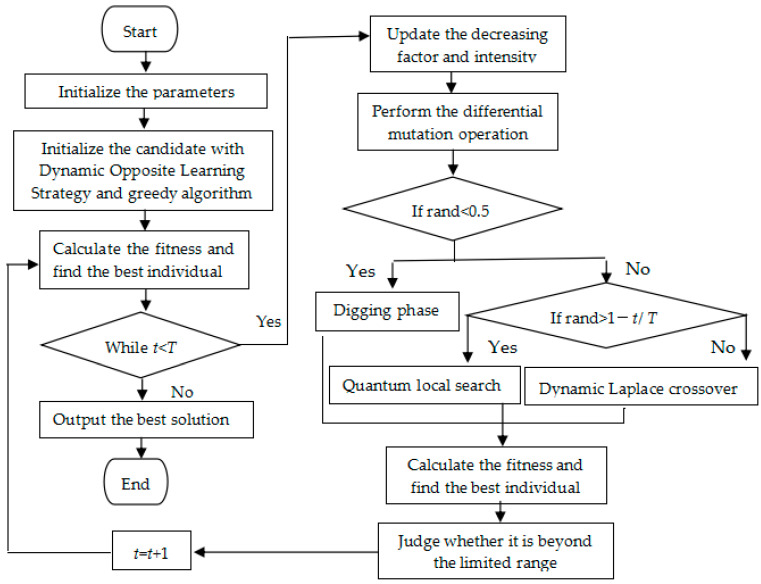
Flowchart for the proposed EHBA optimization algorithm.

For the sake of expressing the EHBA more clearly, the pseudo-code of EHBA is listed in Algorithm 1 and Figure 2 gives the flowchart of the EHBA.
**Algorithm 1:** The Proposed EHBAInput: The parameters of HBA such as *β*, *C*, *N*, *Dim*, and maximum iterations *T*.Output: Optimal fitness value.Random InitializationConstruct the new population through dynamic opposite learning strategy. For i = 1 to *N*
**do** *r*_8_ = rand(0,1), *r*_9_ = rand(0,1), For *j* = 1 to *Dim*
**do** 
$P_{i,j}^{Dobl}=P_{i,j}^{Init}\times r_{8}\times (r_{9}\times (Upb_{j}+Lob_{j}-P_{i,j}^{Init}))-P_{i,j}^{Init}$
Check the boundaries.Using greedy algorithm to select the best initial population from 2*N* populationsEvaluate all fitness value ***F***(*P_i_*), *i* = 1, 2, …, *N*. Save best position ***P****_Best_* and ***F****_Best_*.       While (*t* < *T*) do Renew the decreasing factor *α* by Equation (6).         For *i* =1 to *N* doCalculate the intensity *I*_i_ by Equation (4).       Perform differential mutation operation with Equations (11)–(13): For *i* = 1 to *N*
**do** **Perform** mutation by Equation (11); **End** For *i* = 1 to *N* For *j* = 1 to *Dim*
**do** **Perform** crossover by Equation (12); **End**
**End**
**For**
*i* = 1 to *N*
For *j* = 1 to *Dim*
**do** **Perform** selection by Equation (13); **End**
**End**
            If *r* < 0.5 thenReplace the location ***P****_new_* by Equation (8).             Else                Quantum Local Search: **Perform** Equations (14)–(16)              Else                Dynamic Laplace Crossover:                if *r*1 < 1 − *t*/*T* then Renewed the honey badger location with Equation (21). Else Renewed the honey badger location with Equation (22). End if             End ifEvaluate new positionIf ***F****_new_* ≤ ***F***(*P*_i_) then Let *P_i_* = ***P****_new_* and ***F****_i_* = ***F****_new_*. End ifIf ***F****_new_* ≤ ***F****_Best_* then Make ***P****_Best_* = ***P****_new_* and ***F****_Best_* = ***F****_new_*. End if             End For           Verify the honey badger’s boundaries.           Refresh Honey Badger’s location and most best location (P*)        *t* = *t* + 1      End while

### 3.6. The Complexity Analysis

The calculation complexity of EHBA is determined mainly by the following three operations: dynamic opposite learning initialization, fitness evaluation, and population position update. As the primary stage of the algorithm, the initialization stage is executed only once at the beginning, while the other two steps are performed in each iteration cycle. The complexity is calculated with a default population size of *N*, the iteration period is *T*, and the dimension is defined *D*.

The calculation complexity of the HBA is of *O*(*TND*). The computational complexity of initialization for dynamic directional learning is *O*(*2N*). Quantum local search and Laplace crossover replace the honey harvesting stage of the original algorithm HBA, which is only an order of magnitude operation with a constant multiple *c*, *O*(c*TND*). Constants have little effect on large *O*. In summary, the total computational complexity of EHBA is *O*(c*TND + 2N*). 

## 4. Numerical Experiment and Analysis Results

In this section, we test the performance on the CEC2017, CEC2020, and CEC2022 test sets for demonstrating the effectiveness of the EHBA. CEC2017 contains 30 single objective optimization functions, which is a very classic functions set and also the most capable function set for postgraduate intelligent algorithm optimization ability. The F2 function in the CEC2017 was later removed, as officially declared. The optimization function test kit for CEC2020 includes 10 benchmark problems, which are actually a combination of functions selected from CEC2014 and CEC2017. CEC2022 was also selected from the 2017 and 2014 function sets and is the latest test set for algorithm performance testing. These three test sets contain different uni-modal, multi-modal, hybrid, and composition functions, which can better measure the performance of the new EHBA method. 

In this study, the experiment was run in a Windows 10 (64 bit) environment using Intel (R) core (TM) i5-6500 processors, 3.2 GHz, and 8 GB of main memory. EHBA was implemented in MATLAB R2019a to ensure the fairness of the algorithm. 

The results were compared with the algorithm mentioned in the introduction containing SHO [30], AOA [35], WOA [28], MFO [36], HBA [37], TSA [32], SCA [33], GWO [27], etc., to verify the efficiency of the EHBA when evaluating test problems. These algorithms not only include the earliest classical algorithms proposed, but also include algorithms with better applicability and performance in recent years, which can better reflect the superiority of EHBA in this paper.

Parameter settings for all comparison algorithms should be consistent with those in the various literature; see Table 2. The maximum number of iterations *T* and population size *N* for all methods are 1000 and 30, respectively.

The randomness of meta-heuristics leads to unreliable results from a single run. To ensure a fair comparison, all procedures are performed 30 times independently. Usually, the average accuracy (Mean), standard deviation (Std), best value (Min), worst value (Max), Rank, and Wilcoxon’s rank sum test are selected as the evaluation criteria, which best highlight the effectiveness and feasibility of the algorithm. Here, “+” denotes that the results of other methods are superior to EHBA, “—” defines the number of functions that underperform in EHBA, “=” means that there is no significant difference between EHBA and other methods. Also shown in bold are the minimum values obtained by the eight algorithms listed above. Rank represents the ranking result of the average value of different algorithm. The lower the rank, the better the performance of the algorithm in terms of precision and stability.

### 4.1. Experiment and Analysis on the CEC2017 Test Set

The test function CEC2017 contains 29 functions that are often used to test the effectiveness of algorithms, with at least half being challenging mixed and combined functions [50]. Table 3 presents the test results between EHBA and the other eight methods on the CEC2017. The bold data in the table represent the optimal average data among all the comparison algorithms. In addition, Table 4 shows the Wilcoxon rank sum test results of eight comparative algorithms at a significance level of 0.05.

From Table 3, we can observe that the average rank of EHBA is 1.2069, at the head of their league. The overall solution results of the EHBA are better. Observing the bold data, EHBA achieved smaller values on 86% of the test functions, which were distributed across various functions (uni-modal, multi-modal, mixed, and combined). However, HBA and GWO achieved smaller values on 3 and 1 functions, respectively. Therefore, the number of smaller values obtained by EHBA was much better than that obtained by other comparison algorithms. It is visible that the local quantum search and dynamic Laplacian crossover strategy have improved the effective searching capabilities of the HBA in seeking the best solution. The EHBA can find superior solutions with higher convergence speed based on the original algorithm.

Based on the *p*-value results presented in Table 4, due to the fact that many values are the same, for the convenience of observation, “—” represents the same value 6.79562 × 10^−8^. Combined with Table 3, the final results show that the number of functions of the comparison method that are superior/similar/inferior to EHBA are 3/13/13, 0/12/17, 0/0/29, 0/0/29, 0/0/29, 0/0/29, 1/4/24, and 0/1/28, respectively. It is possible to see that the better HBA in the comparison algorithm outperforms EHBA on 3 test functions, and is inferior to EHBA on 13 test functions. Secondly, the second best GWO in the comparison algorithm outperforms EHBA on function F10, and is inferior to EHBA on 24 test functions. Therefore, overall, the performance of EHBA outperforms the comparison algorithm. Overall, from CEC 2017 test functions, the performance of EHBA is superior to the other eight comparative algorithms. Thus, the experimental results show that the proposed algorithm can effectively solve the CEC2017.

**Figure 3:**
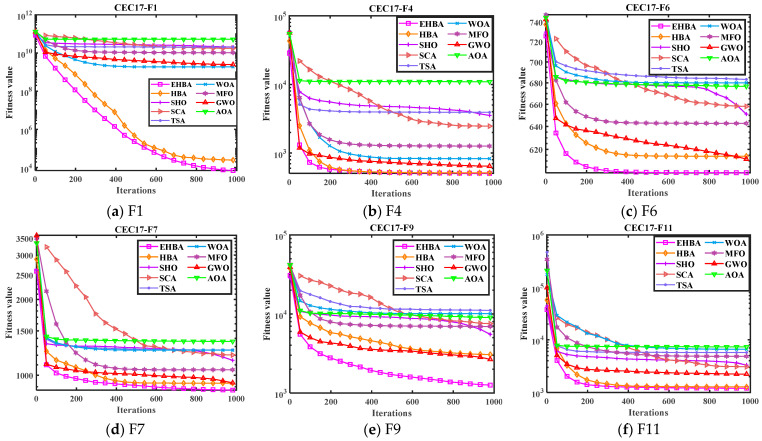

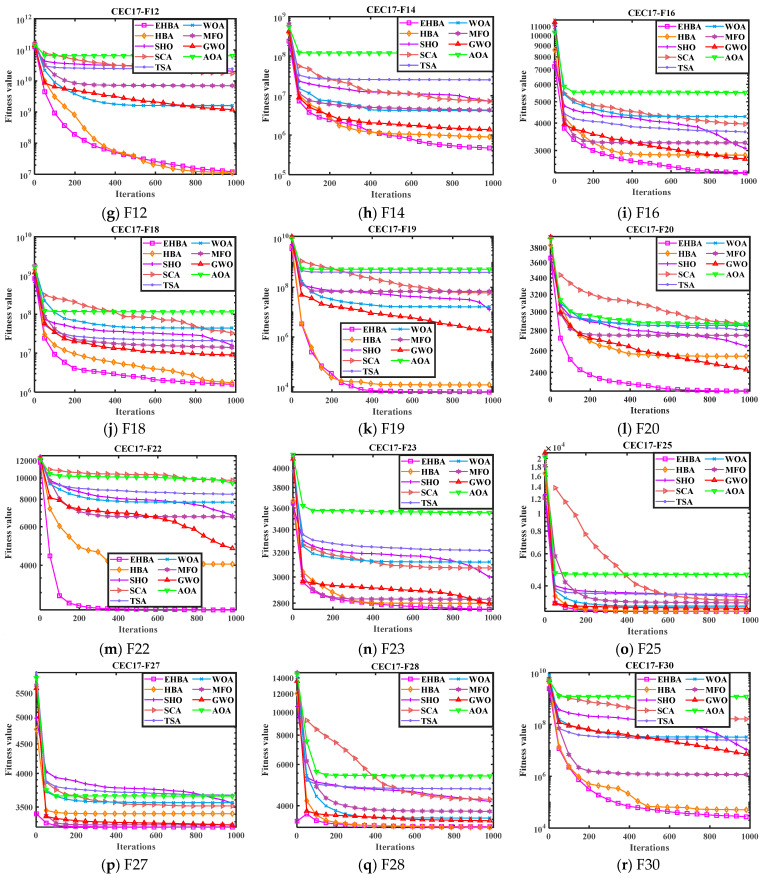
Convergence curves of EHBA and other algorithms on CEC2017 partial test functions.

**Figure 4:**
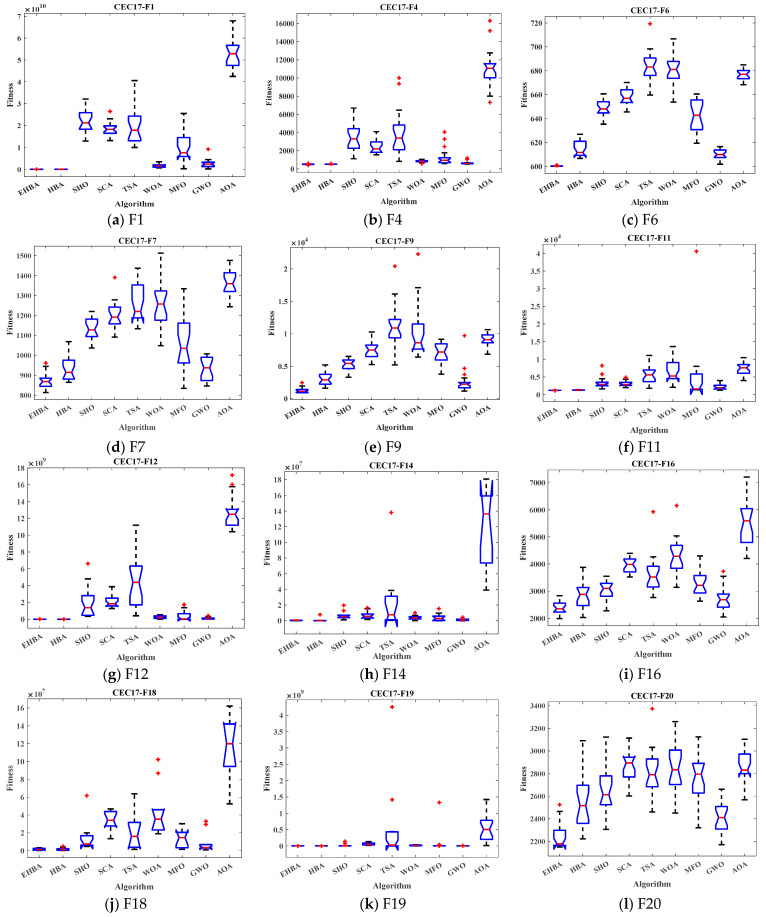

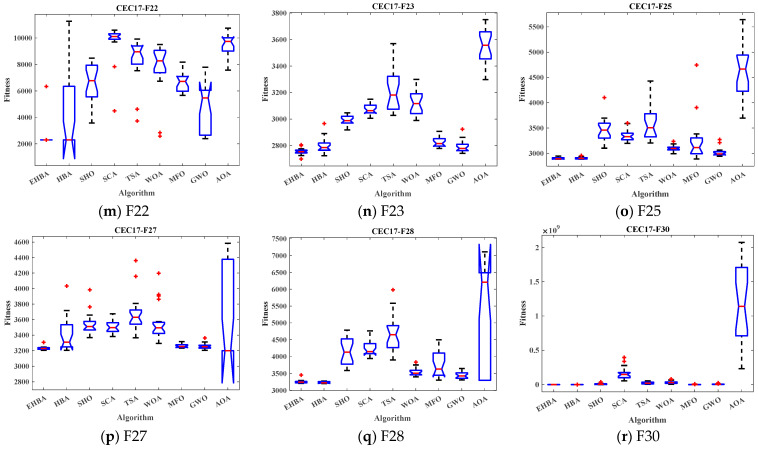
Box plots of EHBA and other algorithms on CEC2017 partial test functions.

In order to display the optimization performance of the EHBA in a more intuitive way, such as its convergence rate, and capability to escape from local optima, and the convenience of observing the trend of curve changes, Figure 3 and Figure 4 show the convergence curves and box plots on some CEC2017 test functions, marking the iterations set as the horizontal axis, the functions use log_10_(*F*) as the vertical axis. From the figure, it can be seen that EHBA can converge to the optimal solution within 1000 iterations continuously, indicating its strong exploration and development capability. The convergence curves indicate that EHBA has a significantly improved convergence accuracy and speed compared to other algorithms. It is evident that the iteration curve of EHBA is able to overcome the local solution in the early stages of iteration and approaches the near-optimal solution; it is close to the optimal solution in the subsequent development period. Specifically, in the convergence curve, EHBA shows more significant convergence effects on F1, F6, F7, F9, F16, F18, F20, F22, and F30, mainly because EHBA has the ability to jump out of the local solution and find the optimal position quickly.

Box plot analysis shows the distribution of the data and helps to understand the distribution of the results. Figure 4 shows the box plot of the results of the EHBA algorithm and other recent optimization algorithms. The box plot is an excellent display showing the distribution of the data based on the quartiles. The red lines of EHBA and HBA show the lowest median, with EHBA being more pronounced. The narrow quartile range of the results obtained by EHBA indicates that the distribution of the obtained solutions is more clustered than other algorithms, and there are few outliers, further demonstrating the stability of EHBA due to the incorporation of the improved strategy. Overall, EHBA is a competitive algorithm that deserves further exploration in practical engineering applications.

### 4.2. Experiment and Analysis on the CEC2020 Test Set

#### 4.2.1. The Ablation Experiments of EHBA

**Figure 5:**
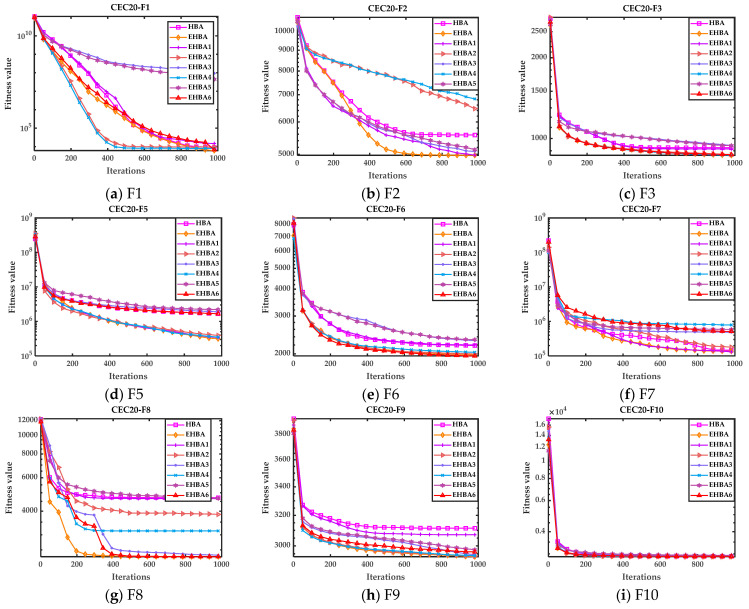
Convergence curves of incomplete algorithms on CEC2020.

In order to verify the effectiveness of the different strategies of EHBA, EHBA is compared with its six incomplete algorithms and HBA. The incomplete algorithms include the dynamic opposite learning strategy, differential mutation strategy, quantum local search, or dynamic Laplace crossover corresponding to EHBA1, EHBA2, EHBA3, and selecting the combination strategies EHBA4 (the dynamic opposite learning strategy and differential mutation strategy), EHBA5 (the dynamic opposite learning strategy and quantum local search or dynamic Laplace crossover strategy), or EHBA6 (the differential mutation strategy and quantum local search or dynamic Laplace crossover strategy) to evaluate their impact on convergence speed and accuracy. Due to article space constraints, this paper only gives the convergence curves of some test sets on the CEC2020 in Figure 5.

F4 in CEC2020 has almost the same convergence accuracy and speed for all comparison algorithms, and has been recombined into a curve. Therefore, it is not shown here. Figure 5a,d indicate that for functions F1 and F5, EHBA has a slower convergence speed than EHBA2 and EHBA4, but has better convergence accuracy. Figure 5c,f–i indicate that for F7–F10, although EHBA has a slightly faster convergence speed than other algorithms, its convergence accuracy is significantly better than other algorithms. For F2, the convergence speed of EHBA is lower than that of ehba1 and EHBA5 in the early stage of iteration, but in the later stage of iteration, the convergence speed and accuracy are significantly better than other algorithms. In general, every improvement strategy of EHBA is effective and its incomplete algorithms all improve HBA to different degrees in both exploration and exploitation. Overall, applying all strategies has a better convergence effect on HBA than its ablation algorithm, which further proves the effectiveness of the added strategies.

The experimental results show that these four strategies have a certain effect on improving the performance of HBA, especially the quantum local search and dynamic Laplace strategy introduced.

#### 4.2.2. Comparison Experiment between Other HBA Variant Algorithms and EHBA

**Figure 6:**
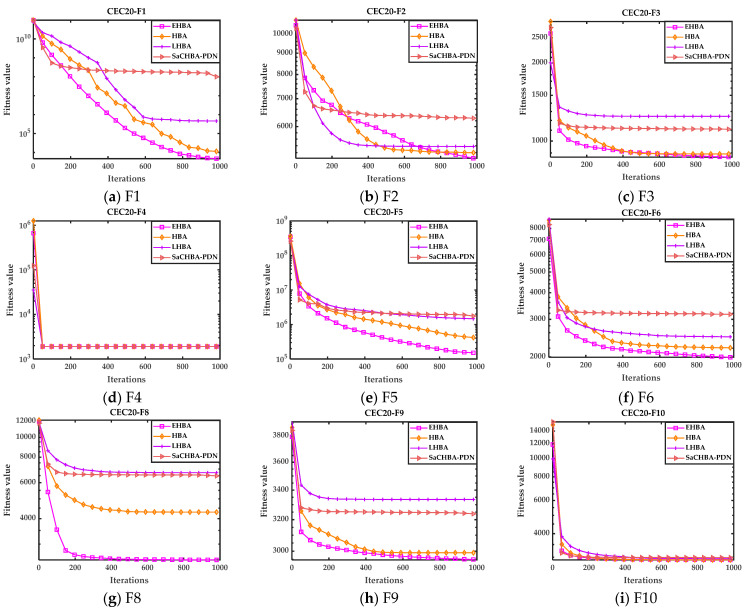
Convergence curves of other HBA variant algorithms and EHBA on CEC2020.

The proposed EHBA are compared with other HBA variants as well to verify its performance. Here, two recently improved variant algorithms have been selected, LHBA [40] and SaCHBA_PDN [41]. To save space, the convergence curve on CEC2020 is presented in Figure 6.

Figure 6 shows that for function F2, although the convergence speed is not as fast as the two variant algorithms LHBA and SaCHBA_PDN in the early iteration, the speed and accuracy are better than LHBA and SaCHBA_PDN in the later stage. Except for F2 in Figure 6, other test functions show that the convergence accuracy and speed of the improved algorithm in this paper are significantly better than other variant algorithms, further indicating that the introduced strategy in this paper has a significant improvement on the original algorithm and has high convergence efficiency. This also demonstrates the effectiveness and high convergence of EHBA proposed in this article.

#### 4.2.3. Comparison Experiments of EHBA and Other Intelligent Algorithms

Similarly, Table 5 presents the comparison results between EHBA and the other eight methods on the test set CEC2020 [51]; it contains 10 functions. At this time, the bold data in the table represent the optimal average data among all the comparison algorithms. In addition, Table 6 lists the Wilcoxon rank sum test results of eight comparative methods under the condition of significance level = 0.05.

From Table 5, we can observe that the average rank of EHBA is 1.4, at the head of their league. The overall solution results of the EHBA algorithm are better. Observing the data in bold, EHBA achieved better values on 80% of the test functions, which were distributed across various functions. However, HBA and GWO achieved smaller values on one function, respectively. Therefore, the number of smaller values obtained by EHBA was much better than that obtained by other comparison algorithms. It is visible that the local quantum search and dynamic Laplacian crossover strategy have improved the searching capabilities of the HBA in seeking the best solution. The EHBA can find superior solutions with higher convergence speed based on the original algorithm.

Based on the *p*-value results presented in Table 6, due to the fact that many values are the same, for the convenience of observation, “—” represents the same value 6.7956 × 10^−8^. Combined with the Table 5, the final results show that the number of functions of each comparison methods better/similar/inferior to EHBA are 1/5/4, 0/2/8, 0/0/10, 0/0/10, 0/1/9, 0/1/9, 3/3/4, 0/1/9, respectively. It is possible to see that the better HBA outperforms EHBA on F5, and is inferior to EHBA on four test functions. Secondly, the second best GWO in the comparison algorithm outperforms EHBA on three test functions, and is superior to EHBA on four test functions. Therefore, overall, the performance of EHBA is good at the other algorithm. Overall, from ten test functions, the performance of EHBA is superior to the other eight comparative algorithms. Thus, the experimental results show that the proposed algorithm can effectively solve the CEC2020.

**Figure 7:**
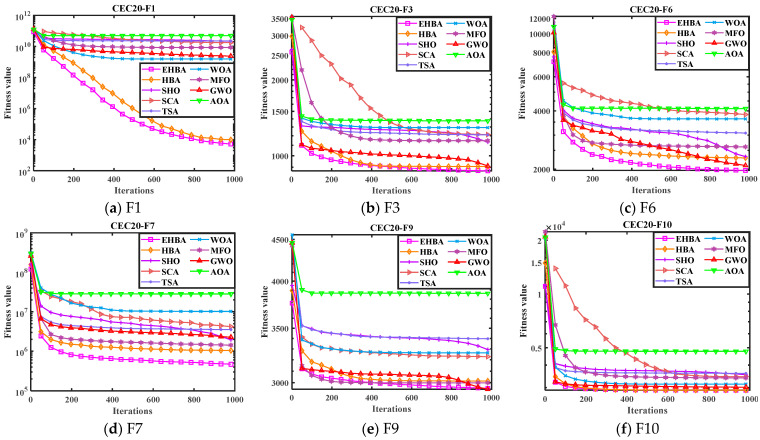
Convergence curves of EHBA and other algorithms on CEC2020 partial test functions.

**Figure 8:**
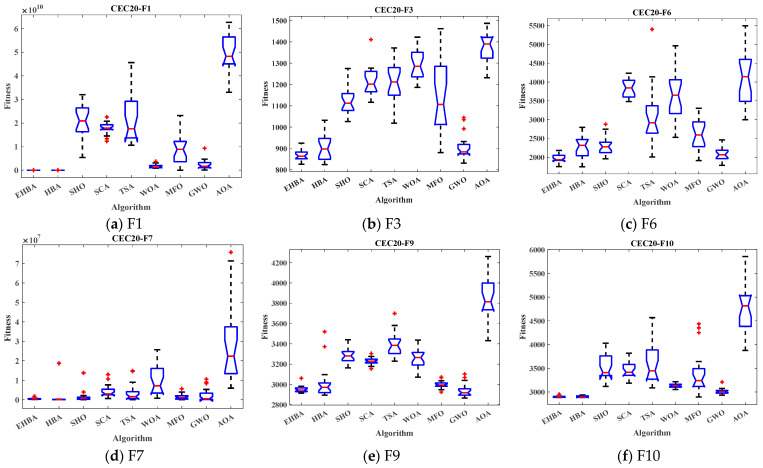
Box plots of EHBA and other algorithms on CEC2020 partial test functions.

Like CEC2017, the convergence curve and box plot are also provided in Figure 7 and Figure 8. The convergence curves indicate that EHBA has significantly improved convergence accuracy and speed compared to other algorithms. It is observed that the iterative curve of EHBA is able to slip away from the local solution in the early stages of iteration and approach the near-optimal solution. It will be close to the optimum solution in the later development phase. Specifically, the convergence curve of EHBA shows more significant convergence effects on F1, F6, and F7. Therefore, the proposed strategy mainly improves the convergence speed of the algorithm in solving the CEC2020 test function, avoiding local stagnation in the optimization process as well as exploration and exploitation capabilities. Overall, EHBA obtains competitive convergence results, and its overall convergence is better than other comparative algorithms.

Figure 8 demonstrated that the box plot of the test function is in line with Table 5. The red lines of EHBA and HBA show the lowest median, with EHBA being more pronounced. The narrow quartile range of the results obtained by EHBA indicates that the distribution of the obtained solutions is more concise than other methods, and there are few outliers, further demonstrating the stability of the EHBA due to the strategy that will be improved. Overall, EHBA is a competitive algorithm that deserves further exploration in practical engineering applications.

### 4.3. Experiment and Analysis on the CEC2022 Test Set

In the same way, Table 7 presents the results between EHBA and other algorithms on the test set CEC2022 [52]; it contains 12 functions. At this time, the bold data in the table represent the optimal average data among all the comparison algorithms. Table 6 also shows the Wilcoxon rank sum test results of eight comparative algorithms with the significance level of 0.05.

The average rank of EHBA is 1.25 from Table 5, top ranking. The overall solution of the EHBA algorithm is better. Observing the bold data, EHBA achieved smaller values on 83% of the test functions, which were distributed across various functions. However, HBA and GWO achieved smaller values on F11 and F4, respectively. Therefore, the number of smaller values obtained by EHBA was much better than that obtained by other comparison algorithms. It is possible to see that the local quantum search and dynamic Laplacian crossover strategy have improved the effective searching capabilities of the HBA in seeking the best solution. The EHBA can find superior solutions with a higher convergence speed based on the original algorithm.

Based on the *p*-value results presented in Table 8. Due to the fact that many values are the same, for the convenience of observation, “—” represents the same value 6.79562 × 10^−8^. Combined with Table 7, the final results show that the number of functions superior/similar/inferior to EHBA are 1/4/7, 0/0/12, 0/0/12, 0/0/12, 0/0/12, 1/1/10, 0/2/10, 0/1/11, respectively. It is possible to observe that the better HBA outperforms EHBA on one test function, and is inferior to EHBA on seven test functions. Secondly, the second best MFO in the comparison algorithm outperforms EHBA on one test function, and is not good at EHBA on ten test functions. Therefore, overall, the performance of EHBA is better than the comparison algorithm. Overall, looking at the 12 test functions, the performance of EHBA is superior to the other 8 comparative algorithms. Thus, the experimental results show that the proposed algorithm can effectively solve the CEC2022.

**Figure 9:**
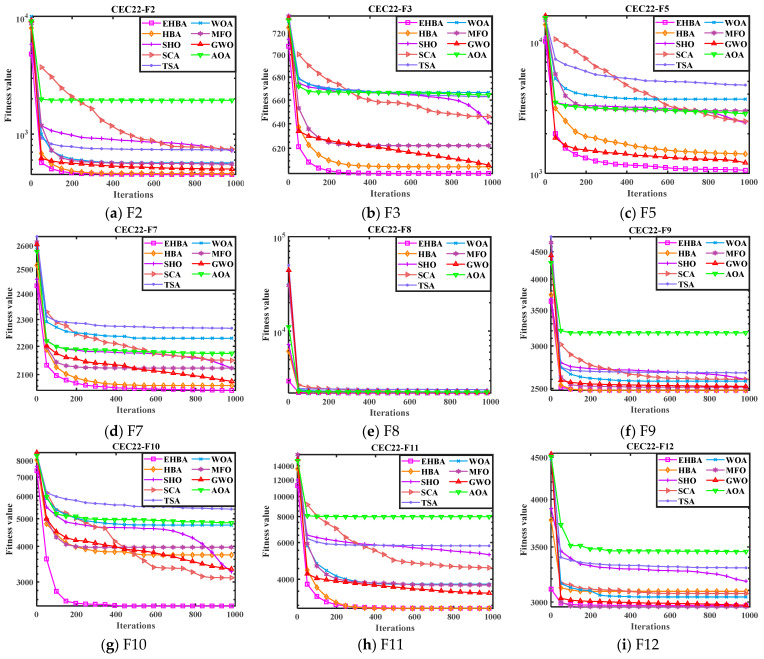
Convergence curves of EHBA and other algorithms on CEC2022 partial test functions.

Like CEC2020, Figure 9 provided the convergence curve IYDSE compared to other meta-heuristics. From the results in the figure, the convergence curves indicate that EHBA has significantly improved convergence accuracy and speed compared to other algorithms. The iterative curve of EHBA can avoid the local solution in the early stages of iteration and converge to the approximate optimal solution. It will be found close to the optimum solution in the subsequent development phase. Specifically, the convergence curve of EHBA shows more significant convergence effects on F3, F5, and F10. Therefore, the proposed strategy mainly improves the convergence speed of the algorithm in solving the CEC2022 test function, avoiding local stagnation in the optimization process as well as exploration and exploitation capabilities.

**Figure 10:**
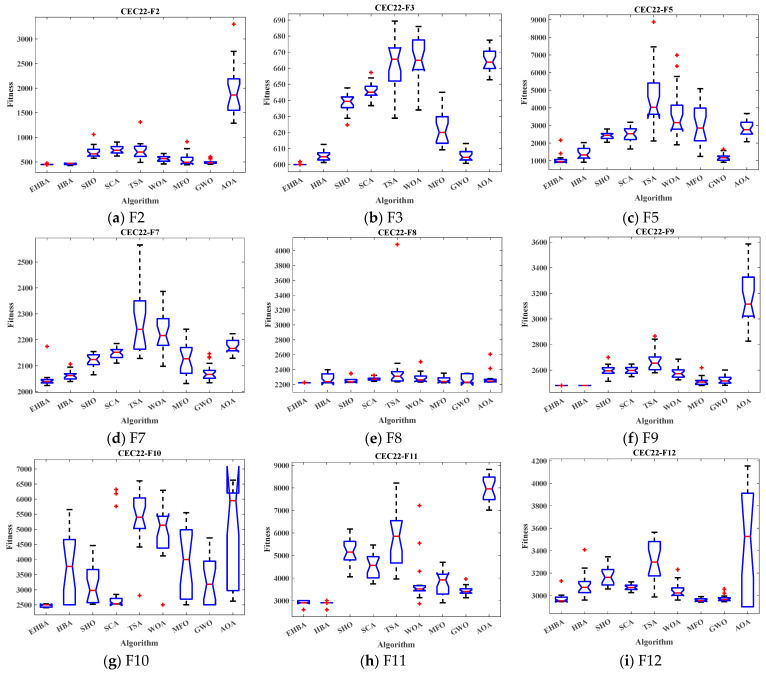
Box plots of EHBA and other algorithms on CEC2022 partial test functions.

The box plot of the test function in Figure 10, the compact box plot, indicates strong data consistency. The red lines of EHBA and HBA show the lowest median, with EHBA being more pronounced. The narrow quartile range of the results obtained by EHBA indicates that the distribution of the obtained solutions is tighter. What is more, there are few outliers, which is also proof of the stability of the EHBA. EHBA has a thinner box plot compared to other algorithms, which indicates the improved performance of the HBA due to the incorporation of the improved strategy. Overall, EHBA is a competitive algorithm that deserves further exploration in practical engineering applications.

In summary, we can see that the EHBA has good convergence, stability, and effectiveness, which provides a solid foundation for solving practical problems.

## 5. The Application of EHBA in Engineering Design Issues

To verify its ability to solve practical problems, the EHBA was used to solve three practical engineering design problems. Before using each algorithm to solve practical engineering optimization problems, a penalty function [53] was used to transform the constrained problem into an unconstrained problem.

### 5.1. Welding Beam Design Issues

**Figure 11:**
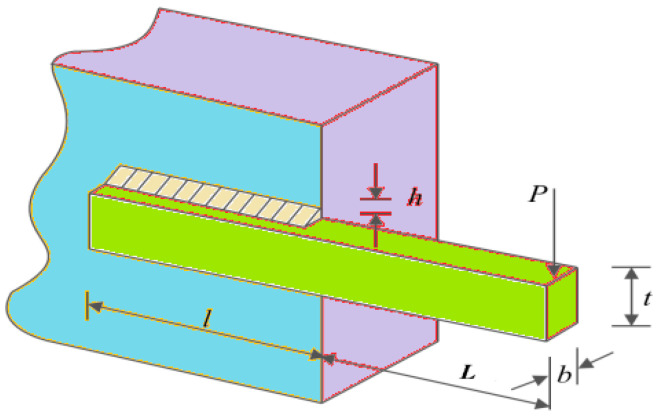
Schematic view of welded beam problem.

Designing welded beams with the lowest manufacturing cost is an effective way to achieve green manufacturing [26]; the schematic view can see the Figure 11. Notably, thickness (*b*), length *(l*), height (*t*) of the electrode, and weld thickness (*h*) are defined as the four optimize variables. At the same time, a load was imposed on the top of the reinforcement; this will result in seven violated constraints, as detailed in Equation (24). Let $\gamma ={\left[h,l,t,b\right]}^{T}={\left[\gamma_{1},\gamma_{2},\gamma_{3},\gamma_{4}\right]}^{T}$ , the formula expression of its mathematical model can be seen in Equation (23). The meanings of relevant variables can be found in reference [26].

(23) $$\mathrm{Min}\;F(\gamma )=1.10471\gamma_{1}^{2}\gamma_{2}+0.04811\gamma_{3}\gamma_{4}\left(14.0+\gamma_{2}\right).$$

The constraint conditions are listed in Equation (24).

(24) $$\{\begin{array}{l} s_{1}\left(\gamma \right)=\tau \left(\gamma \right)-\tau_{\max}\leq 0,g_{2}\left(\gamma \right)=\sigma \left(\gamma \right)-\sigma_{\max}\leq 0,\\ s_{3}\left(\gamma \right)=x_{1}-x_{4}\leq 0,s_{4}\left(\gamma \right)=0.1047\gamma_{1}^{2}+0.04811\gamma_{3}\gamma_{4}\left(14+\gamma_{2}\right)-5\leq 0,\\ s_{5}\left(\gamma \right)=0.125-x_{1}\leq 0,s_{6}\left(\gamma \right)=\delta \left(\gamma \right)-0.25\leq 0,s_{7}\left(\gamma \right)=P-Pc\left(\gamma \right)\leq 0,\\ M=P\left(L+\gamma_{2}/2\right),R=\sqrt{\gamma_{2}^{2}/4+{\left(\left(\gamma_{1}+\gamma_{2}\right)/2\right)}^{2}},\delta \left(\gamma \right)=6PL^{3}/E\gamma_{3}^{2}\gamma_{4},\\ J=2\sqrt{2}\gamma_{1}\gamma_{2}\left(\gamma_{2}^{2}/4+{\left(\left(\gamma_{1}+\gamma_{2}\right)/2\right)}^{2}\right),\sigma \left(\gamma \right)=6PL/\gamma_{4}\gamma_{3}^{2},\\ Pc\left(\gamma \right)=4.013E\sqrt{\gamma_{3}^{2}\gamma_{4}^{6}/36}/L^{2}\left(1-\gamma_{3}/2L\sqrt{E/4G}\right),\\ \tau \left(\gamma \right)=\sqrt{{\left(\tau^{\prime }\right)}^{2}+2\tau^{\prime }\tau^{\prime \prime }\left(\gamma_{2}/2R\right)+{\left(\tau^{\prime \prime }\right)}^{2}},\tau^{\prime }=P/\sqrt{2}\gamma_{1}\gamma_{2},\tau^{\prime \prime }=MR/J. \end{array}$$

The range of variable values is given below, in Equation (25).

(25) $$0.1\leq \gamma_{1},\gamma_{4}\leq 2,\;0.1\leq \gamma_{1},\gamma_{4}\leq 10.$$

**Figure 12:**
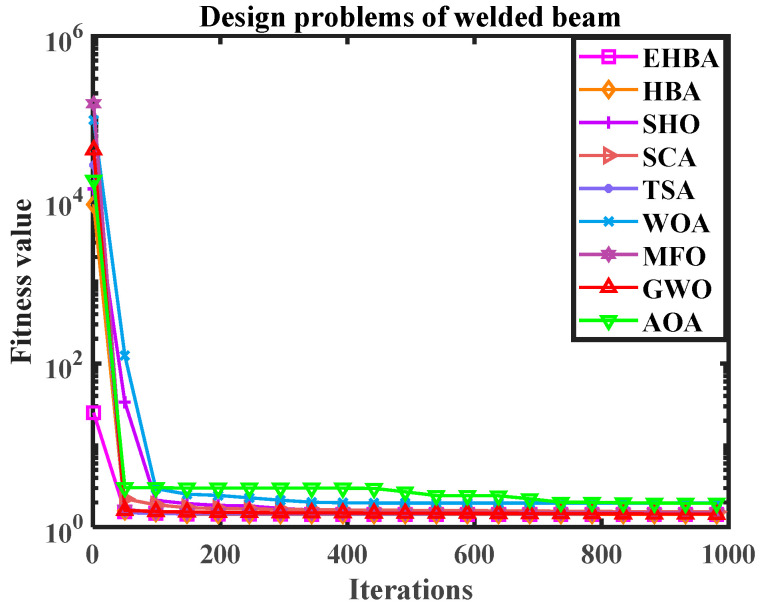
The convergence curve diagram (Design problems of welded beam).

Utilizing EHBA and HBA, SHO, SCA, TSA, WOA, MFO, GWO, and AOA to solve welding beam design problems, Table 9 shows the mean, standard deviation, worst case, and best values independently calculated 20 times in solving the welding beam design problem. Table 10 summarizes the best results in terms of the best results generated by the above algorithms. Simultaneously, the algorithm’s convergence curve diagram is provided in Figure 12; the vertical axis is the logarithm of numerical results, which indicates the efficiency of the EHBA developed in this paper.

From the data analysis in the table, the objective fitness values acquired by MFO and HBA are the same and smaller, meaning that they have high solving precision. Observing the bold data, it is clear that EHBA has performed well on the fourth indicator, with small optimal values, worst values, average values, and standard deviations. Therefore, overall, EHBA has high accuracy in solving this problem and the solution results are relatively stable.

### 5.2. Vehicle Side Impact Design Issues

The goal of the car side impact design problem is to minimize the weight of the car. According to the mathematical model of car side impact established in reference [54], this problem has 11 design elements $\gamma =[\gamma_{1},\gamma_{2},\cdots ,\gamma_{10},\gamma_{11}]$ ; the mathematical expressions of the objective problem is below.

(26) $$\mathrm{Min}\;F(\gamma )=1.98+4.90\gamma_{1}+6.67\gamma_{2}+6.98\gamma_{3}+4.01\gamma_{4}+1.78\gamma_{5}+2.73\gamma_{7}.$$

The constraint conditions that the objective function needs to meet are shown in Equation (27).

(27) $$\left\{ \begin{array}{l} s_{1}(\gamma )=1.16-0.3717\gamma_{2}\gamma_{4}-0.00931\gamma_{2}\gamma_{10}-0.484\gamma_{3}\gamma_{9}+0.01343\gamma_{6}\gamma_{10}-1\leq 0,\\ s_{2}(\gamma )=0.261-0.0159\gamma_{1}\gamma_{2}-0.188\gamma_{1}\gamma_{8}-0.019\gamma_{2}\gamma_{7}+0.0144\gamma_{3}\gamma_{5}+0.0008757\gamma_{5}\gamma_{10}\\ \;+0.08045\gamma_{6}\gamma_{9}+0.00139\gamma_{8}\gamma_{11}+0.00001575\gamma_{10}\gamma_{11}-0.32\leq 0,\\ s_{3}(\gamma )=0.214+0.00817\gamma_{5}-0.131\gamma_{1}\gamma_{8}-0.0704\gamma_{1}\gamma_{9}+0.03099\gamma_{2}\gamma_{6}-0.018\gamma_{2}\gamma_{7}+0.0208\gamma_{3}\gamma_{8}\\ \;+0.121\gamma_{3}\gamma_{9}-0.00364\gamma_{5}\gamma_{6}+0.0007715\gamma_{5}\gamma_{10}-0.0005354\gamma_{6}\gamma_{10}+0.00121\gamma_{8}\gamma_{11}-0.32\leq 0,\\ s_{4}(\gamma )=0.74-0.061\gamma_{2}-0.163\gamma_{3}\gamma_{8}+0.001232\gamma_{3}\gamma_{10}-0.166x_{7}x_{9}+0.227x_{2}^{2}-0.32\leq 0,\\ s_{5}(\gamma )=28.98+3.818\gamma_{3}-4.2\gamma_{1}\gamma_{2}+0.0207\gamma_{5}\gamma_{10}+6.63\gamma_{6}\gamma_{9}-7.7\gamma_{7}\gamma_{8}+0.32\gamma_{9}\gamma_{10}-32\leq 0,\\ s_{6}(\gamma )=33.86+2.95\gamma_{3}+0.1792\gamma_{10}-5.057\gamma_{1}\gamma_{2}-11\gamma_{2}\gamma_{8}-0.0215\gamma_{5}\gamma_{10}-9.98\gamma_{7}\gamma_{8}+22\gamma_{8}\gamma_{9}-32\leq 0,\\ s_{7}(\gamma )=46.36-9.9\gamma_{2}-12.9\gamma_{1}\gamma_{8}+0.1107\gamma_{3}\gamma_{10}-32\leq 0,\\ s_{8}(\gamma )=4.72-0.5\gamma_{4}-0.19\gamma_{2}\gamma_{3}-0.0122\gamma_{4}\gamma_{10}+0.009325\gamma_{6}\gamma_{10}+0.000191\gamma_{11}^{2}-4\leq 0. \end{array}\right.$$

The range of variable values is as follows in Equation (28).

(28) $$\left\{ \begin{array}{l} 0.5\leq \gamma_{1},\gamma_{2},\gamma_{3},\gamma_{4},\gamma_{5},\gamma_{6},\gamma_{7}\leq 1.5,\\ 0.192\leq \gamma_{8},\gamma_{9}\leq 0.345,-30\leq \gamma_{10},\gamma_{11}\leq 30. \end{array}\right.$$

EHBA was implemented to deal with this case and the calculation values were compared with those of HBA, SHO, SCA, TSA, WOA, MFO, GWO, and AOA. Table 11 shows the best values and corresponding variable values for solving the car side impact design problem.

**Figure 13:**
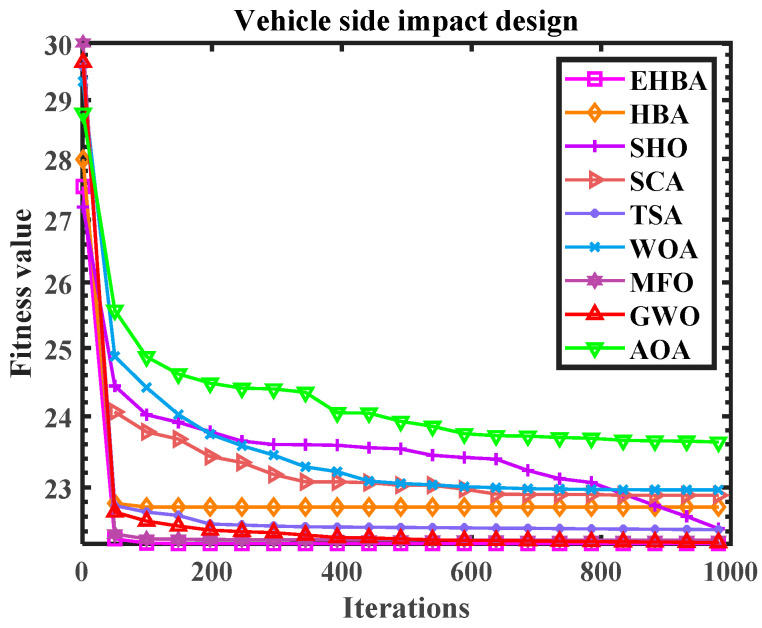
The convergence curve diagram (Vehicle side impact design).

The objective fitness values drawn by MFO, HBA, and EHBA are the same and smaller, making it known that they have high solving accuracy. In addition, Table 12 presents the statistical results running 20 times. The bold data in the table represent the optimal values among all algorithms under each evaluation indicator. Observing the bold data, notably, EHBA has achieved good solving results under the four indicators, with small optimal values, worst values, average values, and standard deviations. Therefore, EHBA has high accuracy and is relatively stable in solving this case. In addition, the convergence curve figure in the above methods is provided in Figure 13; the vertical axis is the logarithm of the numerical solution, which also adds to the proof of the efficiency of EHBA developed in this paper.

### 5.3. Parameter Estimation of Frequency Modulated (FM) Sound Waves

Finding the optimal parameter combination of the six variables for frequency modulation synthesizers is the most critical issue in the problem of frequency modulation sound waves [55]. This is a multi-modal problem. Here, the minimum sum of squared errors between sound waves and target sound waves is defined as the target equation. Let $\gamma =(\gamma_{1},\gamma_{2},\gamma_{3},\gamma_{4},\gamma_{5},\gamma_{6})=(a_{1},\omega_{1},a_{2},\omega_{2},a_{3},\omega_{3})$ , the mathematical description of the problem can be seen in Equation (29).

(29) $$\mathrm{Min}\;F(\gamma )=\sum_{t=0}^{100}{\left[ο(t)-{ο}_{0}(t)\right]}^{2},$$

where

(30) $$\left\{ \begin{array}{l} ο(t)=\gamma_{1}\sin(\gamma_{2}t\theta +\gamma_{3}\sin(\gamma_{4}t\theta +\gamma_{5}\sin(\gamma_{6}t\theta )))\\ {ο}_{0}(t)=\sin(5t\theta -1.5\sin(4.8t\theta +2.0\sin(4.9t\theta ))) \end{array}\right..$$

In Equation (30), θ=2π/100 , *o*(*t*) and *o_0_*(*t*) are the estimating sound waves and target sound wave.

The range of variable values is defined with Equation (31).

(31) $$-6.4\leq a_{1},\omega_{1},a_{2},\omega_{2},a_{3},\omega_{3}\leq 6.35.$$

**Figure 14:**
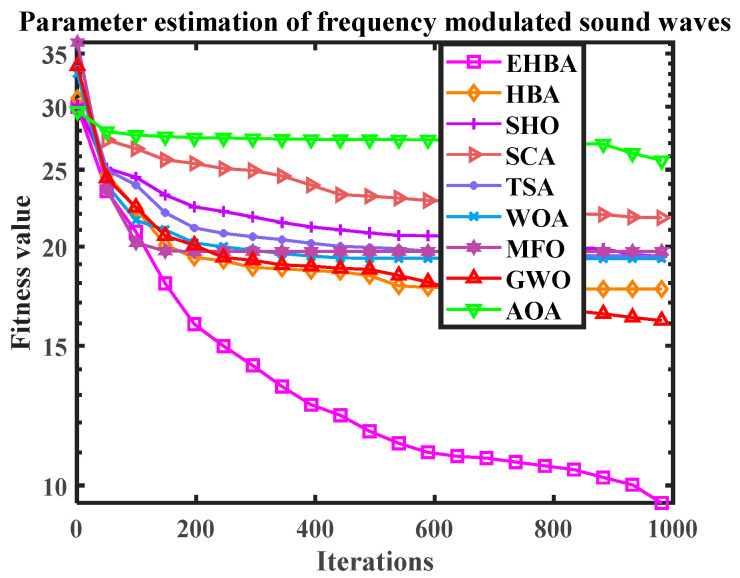
The convergence curve diagram (Parameter estimation of frequency modulated sound waves).

EHBA is applied to deal with the issue of parameter estimation, and the results of EHBA with the original HBA, SHO, SCA, TSA, WOA, MFO, GWO, and AOA are compared. Table 13 lists the best results obtained by all comparison methods. From this, the result obtained by the EHBA are relatively small, indicating that the algorithm has high solving accuracy. In addition, Table 14 presents the statistical results of all methods running 20 times. The bold data are the best value calculated by comparing the algorithms under each indicator (optimal value, worst value, average value, standard deviation). Observing the bold data, it can be seen that although EHBA has a large standard deviation, it can obtain smaller average values, optimal values, and worst values. Therefore, overall, the solution effect of EHBA is relatively good. In addition, the convergence curve diagram for the above methods is provided in Figure 14. The vertical axis is the logarithm of numerical results, which also adds to the proof of the efficiency of EHBA developed in this paper.

## 6. Conclusions and Future Research

A multi-strategy fusion enhanced optimization algorithm (EHBA) is proposed based on the dynamic opposite learning, differential variation and selectively, local quantum search, or dynamic Laplacian crossover operators to address the issues of local optima and slow convergence speed in the HBA. The adoption of a dynamic opposite learning strategy broadens the search area of the population, enhances global search ability, and improves population diversity and the quality of solutions. Differential mutation operation not only enhances the precision and exploration, but also secures the convergence rate of the HBA. Introducing a local quantum search strategy during the honey harvesting stage (development), the local search capabilities are enhanced and the population optimization precision is improved. Alternatively, introducing dynamic Laplacian crossover operators can improve convergence speed, which reduces the probability of EHBA sinking into local optima to a certain extent. Through comparative experiments with other algorithms on the CEC2017, CEC2020, and CEC2022 test sets, along with three engineering examples, EHBA has been verified to have good solving performance compared with other intelligent algorithms and other variant algorithms. From the convergence curve, box plot, and comparative analysis of algorithm performance testing, it will be on display that compared with the other eight comparative methods, EHBA has significantly improved optimization ability and convergence speed, and is expected to prove useful in optimizing problems.

Due to the superiority of EHBA, it may be implemented for multi-objective problems in more scientific research areas, such as robot movement, missile trajectory, image segmentation, predictive modeling, feature selection [56], geometry optimization [57], and engineering design [58,59] in the future. In addition, effective improvements to the original algorithm HBA can not only add different and good strategies, but also integrate other excellent algorithms.

## Figures and Tables

**Table 2:** Parameters setting.

Algorithms	Parameters	Setting Value
HBA, EHBA	Coefficient of the logarithmic spiral shape *β* (the ability of a honey badger to get food)	6
	*C*	2
SHO	Logarithmic helix constant	*u* = 0.05, *v* = 0.05
	Constant parameters *l*	*l* = 0.05
AOA	Constant parameters	*c*1 = 2, *c*2 = 6, *c*3 = 1, *c*4 = 2
WOA	Control parameter *a* Constant parameters *b*	*a* is linear decrease from 2 to 0 *b* = 1
MFO	Shape constant of logarithmic spiral *b*	*b* = 1
TSA	Initial interaction velocity constant *Pmin*, *Pmax*	*Pmin* = 1, *Pmax* = 4
SCA	Constant parameters *a*	*a* = 2
GWO	Control parameter *a*	*a* is linear decrease from 2 to 0

**Table 3:** Comparison results of EHBA and other methods on CEC2017.

F	Index	Algorithms								
		EHBA	HBA	SHO	SCA	TSA	WOA	MFO	GWO	AOA
F1	Ave	**6.940 × 10^3^**	2.116 × 10^4^	2.036 × 10^10^	1.776 × 10^10^	2.043 × 10^10^	1.875 × 10^9^	1.044 × 10^10^	2.469 × 10^9^	5.208 × 10^10^
	Std	6.075 × 10^3^	5.366 × 10^4^	6.035 × 10^9^	2.884 × 10^9^	5.480 × 10^9^	1.367 × 10^9^	8.340 × 10^9^	1.791 × 10^9^	7.679 × 10^9^
	Best	4.300 × 10^2^	9.124 × 10^2^	6.912 × 10^9^	1.246 × 10^10^	6.148 × 10^9^	6.860 × 10^8^	1.824 × 10^9^	7.948 × 10^8^	4.107 × 10^10^
	Rank	1	2	7	6	8	3	5	4	9
F3	Ave	2.908 × 10^4^	**1.885 × 10^4^**	6.359 × 10^4^	6.253 × 10^4^	4.720 × 10^4^	2.536 × 10^5^	1.388 × 10^5^	5.102 × 10^4^	5.539 × 10^4^
	Std	6.627 × 10^3^	5.071 × 10^3^	9.618 × 10^3^	1.100 × 10^4^	9.274 × 10^3^	7.388 × 10^4^	6.077 × 10^4^	1.381 × 10^4^	9.598 × 10^3^
	Best	1.723 × 10^4^	1.077 × 10^4^	4.048 × 10^4^	4.732 × 10^4^	2.599 × 10^4^	1.688 × 10^5^	4.195 × 10^4^	1.461 × 10^4^	3.291 × 10^4^
	Rank	2	1	7	6	3	9	8	4	5
F4	Ave	**5.049 × 10^2^**	5.151 × 10^2^	3.487 × 10^3^	2.466 × 10^3^	3.914 × 10^3^	8.298 × 10^2^	1.263 × 10^3^	6.419 × 10^2^	1.096 × 10^4^
	Std	3.796 × 10^1^	2.728 × 10^1^	1.636 × 10^3^	8.131 × 10^2^	2.526 × 10^3^	1.009 × 10^2^	9.391 × 10^2^	1.665 × 10^2^	2.162 × 10^3^
	Best	4.046 × 10^2^	4.733 × 10^2^	1.118 × 10^3^	1.545 × 10^3^	8.272 × 10^2^	5.865 × 10^2^	5.877 × 10^2^	5.272 × 10^2^	7.314 × 10^3^
	Rank	1	2	7	6	8	4	5	3	9
F5	Ave	**6.103 × 10^2^**	6.216 × 10^2^	7.268 × 10^2^	8.185 × 10^2^	8.335 × 10^2^	8.309 × 10^2^	7.045 × 10^2^	6.204 × 10^2^	8.680 × 10^2^
	Std	1.664 × 10^1^	2.609 × 10^1^	2.710 × 10^1^	2.758 × 10^1^	4.902 × 10^1^	5.581 × 10^1^	3.487 × 10^1^	4.249 × 10^1^	2.702 × 10^1^
	Best	5.853 × 10^2^	5.657 × 10^2^	6.912 × 10^2^	7.852 × 10^2^	7.145 × 10^2^	6.699 × 10^2^	6.440 × 10^2^	5.659 × 10^2^	8.008 × 10^2^
	Rank	1	3	5	6	8	7	4	2	9
F6	Ave	**6.002 × 10^2^**	6.141 × 10^2^	6.485 × 10^2^	6.581 × 10^2^	6.833 × 10^2^	6.801 × 10^2^	6.428 × 10^2^	6.104 × 10^2^	6.768 × 10^2^
	Std	2.683 × 10^−1^	6.140 × 10^0^	6.153 × 10^0^	6.172 × 10^0^	1.378 × 10^1^	1.205 × 10^1^	1.368 × 10^1^	4.147 × 10^0^	4.811 × 10^0^
	Best	6.000 × 10^2^	6.066 × 10^2^	6.352 × 10^2^	6.455 × 10^2^	6.597 × 10^2^	6.537 × 10^2^	6.194 × 10^2^	6.018 × 10^2^	6.682 × 10^2^
	Rank	1	3	5	6	9	8	4	2	7
F7	Ave	**8.715 × 10^2^**	9.302 × 10^2^	1.131 × 10^3^	1.202 × 10^3^	1.258 × 10^3^	1.256 × 10^3^	1.052 × 10^3^	9.302 × 10^2^	1.362 × 10^3^
	Std	3.822 × 10^1^	5.782 × 10^1^	5.359 × 10^1^	6.706 × 10^1^	1.000 × 10^2^	1.216 × 10^2^	1.466 × 10^2^	5.838 × 10^1^	5.943 × 10^1^
	Best	8.130 × 10^2^	8.640 × 10^2^	1.036 × 10^3^	1.090 × 10^3^	1.132 × 10^3^	1.048 × 10^3^	8.339 × 10^2^	8.459 × 10^2^	1.243 × 10^3^
	Rank	1	3	5	6	8	7	4	2	9
F8	Ave	**8.985 × 10^2^**	9.036 × 10^2^	9.781 × 10^2^	1.081 × 10^3^	1.111 × 10^3^	1.059 × 10^3^	1.016 × 10^3^	8.991 × 10^2^	1.100 × 10^3^
	Std	2.029 × 10^1^	2.444 × 10^1^	2.901 × 10^1^	2.194 × 10^1^	5.049 × 10^1^	5.459 × 10^1^	5.290 × 10^1^	2.235 × 10^1^	2.551 × 10^1^
	Best	8.701 × 10^2^	8.557 × 10^2^	9.206 × 10^2^	1.040 × 10^3^	1.031 × 10^3^	9.860 × 10^2^	9.466 × 10^2^	8.705 × 10^2^	1.049 × 10^3^
	Rank	1	3	4	7	9	6	5	2	8
F9	Ave	**1.235 × 10^2^**	3.043 × 10^3^	5.260 × 10^3^	7.498 × 10^3^	1.105 × 10^4^	1.007 × 10^4^	6.909 × 10^3^	2.599 × 10^3^	9.035 × 10^3^
	Std	4.100 × 10^2^	9.862 × 10^2^	8.810 × 10^2^	1.349 × 10^3^	3.400 × 10^3^	3.877 × 10^3^	1.678 × 10^3^	1.874 × 10^3^	9.305 × 10^2^
	Best	9.054 × 10^2^	1.623 × 10^3^	3.284 × 10^3^	5.246 × 10^3^	5.192 × 10^3^	6.380 × 10^3^	3.775 × 10^3^	1.175 × 10^3^	6.816 × 10^3^
	Rank	1	3	4	6	9	8	5	2	7
F10	Ave	**4.970 × 10^3^**	5.513 × 10^3^	5.693 × 10^3^	8.736 × 10^3^	7.183 × 10^3^	7.257 × 10^3^	5.657 × 10^3^	4.575 × 10^3^	8.405 × 10^3^
	Std	6.279 × 10^2^	1.498 × 10^3^	5.170 × 10^2^	2.679 × 10^2^	6.318 × 10^2^	8.110 × 10^2^	6.857 × 10^2^	9.121 × 10^2^	3.467 × 10^2^
	Best	2.817 × 10^3^	3.856 × 10^3^	4.769 × 10^3^	8.208 × 10^3^	5.945 × 10^3^	5.750 × 10^3^	4.122 × 10^3^	3.673 × 10^3^	7.486 × 10^3^
	Rank	2	3	5	9	6	7	4	1	8
F11	Ave	**1.201 × 10^3^**	1.274 × 10^3^	3.282 × 10^3^	3.080 × 10^3^	5.717 × 10^3^	6.606 × 10^3^	4.853 × 10^3^	2.233 × 10^3^	7.360 × 10^3^
	Std	3.024 × 10^1^	5.679 × 10^1^	1.494 × 10^3^	7.678 × 10^2^	2.695 × 10^3^	3.322 × 10^3^	8.710 × 10^3^	9.032 × 10^2^	1.705 × 10^3^
	Best	1.132 × 10^3^	1.187 × 10^3^	1.620 × 10^3^	1.977 × 10^3^	1.772 × 10^3^	2.088 × 10^3^	1.375 × 10^3^	1.275 × 10^3^	3.978 × 10^3^
	Rank	1	2	5	4	7	8	6	3	9
F12	Ave	1.169 × 10^7^	**1.096 × 10^7^**	1.983 × 10^10^	1.762 × 10^10^	2.455 × 10^10^	1.623 × 10^9^	7.032 × 10^9^	1.186 × 10^9^	6.502 × 10^10^
	Std	6.141 × 10^6^	9.818 × 10^6^	7.132 × 10^9^	3.949 × 10^9^	1.384 × 10^10^	5.255 × 10^8^	5.918 × 10^9^	1.111 × 10^9^	9.469 × 10^9^
	Best	3.678 × 10^6^	3.699 × 10^6^	8.820 × 10^9^	1.406 × 10^10^	8.337 × 10^9^	6.302 × 10^8^	1.132 × 10^9^	1.201 × 10^8^	5.259 × 10^10^
	Rank	2	1	7	6	8	4	5	3	9
F13	Ave	**2.862 × 10^4^**	4.082× 10^4^	4.596 × 10^8^	8.223 × 10^8^	3.208 × 10^9^	2.150 × 10^6^	7.182 × 10^7^	1.436 × 10^7^	5.248 × 10^9^
	Std	6.761 × 10^4^	3.591× 10^4^	1.181 × 10^9^	2.120 × 10^8^	4.547 × 10^9^	2.336 × 10^6^	3.031 × 10^8^	3.725 × 10^7^	1.977 × 10^9^
	Best	2.462 × 10^3^	7.882 × 10^3^	8.005 × 10^5^	5.302 × 10^8^	4.076 × 10^7^	2.296 × 10^5^	2.883 × 10^4^	2.902 × 10^4^	1.371 × 10^9^
	Rank	1	2	6	7	8	3	5	4	9
F14	Ave	**4.670 × 10^5^**	9.018 × 10^5^	7.040 × 10^6^	7.230 × 10^6^	2.527 × 10^7^	4.218 × 10^6^	4.315 × 10^6^	1.368 × 10^6^	1.196 × 10^8^
	Std	2.301 × 10^5^	2.423 × 10^6^	5.367 × 10^6^	5.031 × 10^6^	4.207 × 10^7^	2.794 × 10^6^	4.907 × 10^6^	1.205 × 10^6^	5.024 × 10^7^
	Best	1.379 × 10^5^	4.415 × 10^4^	1.256 × 10^6^	1.709 × 10^6^	6.223 × 10^5^	2.136 × 10^5^	1.560 × 10^5^	1.082 × 10^5^	3.903 × 10^7^
	Rank	1	2	6	7	8	4	5	3	9
F15	Ave	**1.124 × 10^4^**	1.65 × 10^4^	2.910 × 10^5^	3.238 × 10^7^	1.373 × 10^8^	3.773 × 10^6^	4.010 × 10^4^	3.399 × 10^6^	2.103 × 10^8^
	Std	1.343 × 10^4^	1.406 × 10^4^	5.303 × 10^5^	2.574 × 10^7^	2.274 × 10^8^	6.591 × 10^6^	2.966 × 10^4^	1.466 × 10^7^	2.340 × 10^8^
	Best	1.836 × 10^3^	3.024 × 10^3^	1.386 × 10^6^	1.754 × 10^6^	9.574 × 10^4^	1.417 × 10^5^	6.000 × 10^3^	2.200 × 10^4^	6.277 × 10^6^
	Rank	1	2	4	7	8	6	3	5	9
F16	Ave	**2.375 × 10^3^**	2.860 × 10^3^	3.054 × 10^3^	3.955 × 10^3^	3.646 × 10^3^	4.286 × 10^3^	3.255 × 10^3^	2.740 × 10^3^	5.506 × 10^3^
	Std	2.289 × 10^2^	4.421 × 10^2^	3.268 × 10^2^	2.684 × 10^2^	6.790 × 10^2^	7.032 × 10^2^	4.167 × 10^2^	4.395 × 10^2^	8.078 × 10^2^
	Best	1.986 × 10^3^	2.026 × 10^3^	2.277 × 10^3^	3.519 × 10^3^	2.767 × 10^3^	3.139 × 10^3^	2.631 × 10^3^	2.047 × 10^3^	4.204 × 10^3^
	Rank	1	3	4	7	6	8	5	2	9
F17	Ave	**1.944 × 10^3^**	2.214 × 10^3^	2.408 × 10^3^	2.672 × 10^3^	2.595 × 10^3^	2.808 × 10^3^	2.602 × 10^3^	2.018 × 10^3^	3.334 × 10^3^
	Std	1.504 × 10^2^	2.166 × 10^2^	2.663 × 10^2^	1.522 × 10^2^	3.417 × 10^2^	2.243 × 10^2^	3.607 × 10^2^	1.093 × 10^2^	4.563 × 10^2^
	Best	1.759 × 10^3^	1.859 × 10^3^	1.886 × 10^3^	2.432 × 10^3^	2.043 × 10^3^	2.439 × 10^3^	1.957 × 10^3^	1.832 × 10^3^	2.615 × 10^3^
	Rank	1	3	4	7	5	8	6	2	9
F18	Ave	**1.572 × 10^6^**	1.701 × 10^6^	1.457 × 10^7^	3.276 × 10^7^	2.070 × 10^7^	4.441 × 10^7^	1.377 × 10^7^	8.983 × 10^6^	1.157 × 10^8^
	Std	1.083 × 10^6^	1.540 × 10^6^	1.733 × 10^7^	1.154 × 10^7^	2.021 × 10^7^	2.813 × 10^7^	1.061 × 10^7^	1.183 × 10^7^	3.164 × 10^7^
	Best	2.608 × 10^5^	2.711 × 10^5^	4.623 × 10^6^	1.346 × 10^7^	1.374 × 10^6^	1.894 × 10^7^	1.344 × 10^6^	7.288 × 10^5^	5.250 × 10^7^
	Rank	1	2	5	7	6	8	4	3	9
F19	Ave	**6.171 × 10^3^**	1.205 × 10^4^	1.056 × 10^7^	5.981 × 10^7^	3.992 × 10^8^	1.648 × 10^7^	6.896 × 10^7^	1.677 × 10^6^	5.337 × 10^8^
	Std	5.556 × 10^3^	1.457 × 10^4^	3.343 × 10^7^	3.563 × 10^7^	9.671 × 10^8^	1.085 × 10^7^	2.969 × 10^8^	2.021 × 10^6^	3.838 × 10^8^
Best	Best	1.936 × 10^3^	2.162 × 10^3^	1.021 × 10^4^	1.918 × 10^7^	1.582 × 10^5^	5.604 × 10^5^	1.109 × 10^4^	8.336 × 10^3^	1.025 × 10^7^
	Rank	1	2	4	6	8	5	7	3	9
F20	Ave	**2.240 × 10^3^**	2.547 × 10^3^	2.638 × 10^3^	2.865 × 10^3^	2.808 × 10^3^	2.853 × 10^3^	2.750 × 10^3^	2.421 × 10^3^	2.868 × 10^3^
	Std	1.131 × 10^2^	2.447 × 10^2^	2.019 × 10^2^	1.332 × 10^2^	2.107 × 10^2^	2.034 × 10^2^	2.176 × 10^2^	1.364 × 10^2^	1.370 × 10^2^
	Best	2.149 × 10^3^	2.222 × 10^3^	2.305 × 10^3^	2.601 × 10^3^	2.460 × 10^3^	2.451 × 10^3^	2.320 × 10^3^	2.171 × 10^3^	2.568 × 10^3^
	Rank	1	3	4	8	6	7	5	2	9
F21	Ave	**2.401 × 10^3^**	2.406 × 10^3^	2.503 × 10^3^	2.591 × 10^3^	2.650 × 10^3^	2.619 × 10^3^	2.498 × 10^3^	2.415 × 10^3^	2.628 × 10^3^
	Std	2.010 × 10^1^	3.290 × 10^1^	2.864 × 10^1^	2.077 × 10^1^	4.877 × 10^1^	6.677 × 10^1^	5.385 × 10^1^	4.413 × 10^1^	2.389 × 10^1^
	Best	2.365 × 10^3^	2.347 × 10^3^	2.455 × 10^3^	2.554 × 10^3^	2.556 × 10^3^	2.538 × 10^3^	2.391 × 10^3^	2.360 × 10^3^	2.583 × 10^3^
	Rank	1	2	5	6	9	7	4	3	8
F22	Ave	**2.502 × 10^3^**	4.053 × 10^3^	6.678 × 10^3^	9.760 × 10^3^	8.438 × 10^3^	7.757 × 10^3^	6.669 × 10^3^	4.752 × 10^3^	9.449 × 10^3^
	Std	9.030 × 10^2^	2.669 × 10^3^	1.420 × 10^3^	1.367 × 10^3^	1.624 × 10^3^	1.927 × 10^3^	7.371 × 10^2^	1.762 × 10^3^	8.479 × 10^2^
	Best	2.300 × 10^3^	2.300 × 10^3^	3.574 × 10^3^	4.487 × 10^3^	3.731 × 10^3^	2.585 × 10^3^	5.660 × 10^3^	2.396 × 10^3^	7.570 × 10^3^
	Rank	1	2	5	9	7	6	4	3	8
F23	Ave	**2.754 × 10^3^**	2.799 × 10^3^	2.987 × 10^3^	3.073 × 10^3^	3.218 × 10^3^	3.121 × 10^3^	2.829 × 10^3^	2.792 × 10^3^	3.556 × 10^3^
	Std	2.395 × 10^1^	5.438 × 10^1^	4.057 × 10^1^	3.885 × 10^1^	1.546 × 10^2^	9.451 × 10^1^	3.803 × 10^1^	4.361 × 10^1^	1.205 × 10^2^
	Best	2.700 × 10^3^	2.724 × 10^3^	2.918 × 10^3^	3.005 × 10^3^	3.028 × 10^3^	2.989 × 10^3^	2.779 × 10^3^	2.741 × 10^3^	3.297 × 10^3^
	Rank	1	3	5	6	8	7	4	2	9
F24	Ave	2.960 × 10^3^	3.086 × 10^3^	3.300 × 10^3^	3.227 × 10^3^	3.365 × 10^3^	3.229 × 10^3^	2.991 × 10^3^	**2.923 × 10^3^**	3.772 × 10^3^
	Std	3.035 × 10^1^	1.867 × 10^2^	7.077 × 10^1^	4.405 × 10^1^	1.219 × 10^2^	1.025 × 10^2^	3.539 × 10^1^	3.966 × 10^1^	1.888 × 10^2^
	Best	2.902 × 10^3^	2.851 × 10^3^	3.195 × 10^3^	3.156 × 10^3^	3.158 × 10^3^	3.060 × 10^3^	2.940 × 10^3^	2.867 × 10^3^	3.467 × 10^3^
	Rank	2	4	7	5	8	6	3	1	9
F25	Ave	**2.901 × 10^3^**	2.905 × 10^3^	3.456 × 10^3^	3.345 × 10^3^	3.593 × 10^3^	3.098 × 10^3^	3.232 × 10^3^	3.015 × 10^3^	4.624 × 10^3^
	Std	1.828 × 10^1^	1.957 × 10^1^	2.252 × 10^2^	1.121 × 10^2^	3.408 × 10^2^	5.812 × 10^1^	4.286 × 10^2^	8.485 × 10^1^	4.476 × 10^2^
	Best	2.884 × 10^3^	2.884 × 10^3^	3.102 × 10^3^	3.196 × 10^3^	3.203 × 10^3^	2.991 × 10^3^	2.888 × 10^3^	2.940 × 10^3^	3.695 × 10^3^
	Rank	1	2	7	6	8	4	5	3	9
F26	Ave	4.555 × 10^3^	**4.504 × 10^3^**	7.303 × 10^3^	7.551 × 10^3^	8.400 × 10^3^	8.546 × 10^3^	6.068 × 10^3^	4.891 × 10^3^	1.029 × 10^4^
	Std	6.324 × 10^2^	1.120 × 10^3^	8.007 × 10^2^	3.042 × 10^2^	1.726 × 10^3^	9.515 × 10^2^	5.199 × 10^2^	4.851 × 10^2^	6.964 × 10^2^
	Best	2.800 × 10^3^	2.811 × 10^3^	5.649 × 10^3^	7.043 × 10^3^	3.796 × 10^3^	7.109 × 10^3^	5.081 × 10^3^	4.087 × 10^3^	9.013 × 10^3^
	Rank	2	1	5	6	7	8	4	3	9
F27	Ave	**3.231 × 10^3^**	3.408 × 10^3^	3.540 × 10^3^	3.514 × 10^3^	3.670 × 10^3^	3.560 × 10^3^	3.261 × 10^3^	3.257 × 10^3^	3.654 × 10^3^
	Std	2.172 × 10^1^	2.140 × 10^2^	1.374 × 10^2^	8.689 × 10^1^	2.326 × 10^2^	2.318 × 10^2^	2.638 × 10^1^	3.699 × 10^1^	6.057 × 10^2^
	Best	3.204 × 10^3^	3.203 × 10^3^	3.368 × 10^3^	3.382 × 10^3^	3.366 × 10^3^	3.293 × 10^3^	3.231 × 10^3^	3.203 × 10^3^	3.200 × 10^3^
	Rank	1	4	6	5	9	7	3	2	8
F28	Ave	3.253 × 10^3^	**3.233 × 10^3^**	4.160 × 10^3^	4.262 × 10^3^	4.721 × 10^3^	3.544 × 10^3^	3.783 × 10^3^	3.451 × 10^3^	5.352 × 10^3^
	Std	5.239 × 10^1^	2.465 × 10^1^	4.107 × 10^2^	2.602 × 10^2^	5.381 × 10^2^	1.141 × 10^2^	3.947 × 10^2^	1.029 × 10^2^	1.571 × 10^3^
	Best	3.206 × 10^3^	3.203 × 10^3^	3.590 × 10^3^	3.942 × 10^3^	3.898 × 10^3^	3.403 × 10^3^	3.304 × 10^3^	3.310 × 10^3^	3.300 × 10^3^
	Rank	2	1	6	7	8	4	5	3	9
F29	Ave	**3.621 × 10^3^**	4.379 × 10^3^	4.350 × 10^3^	5.116 × 10^3^	4.857 × 10^3^	5.207 × 10^3^	4.299 × 10^3^	3.905 × 10^3^	6.297 × 10^3^
	Std	1.801 × 10^2^	8.364 × 10^2^	2.871 × 10^2^	3.841 × 10^2^	4.141 × 10^2^	4.619 × 10^2^	3.061 × 10^2^	2.067 × 10^2^	7.947 × 10^2^
Best	Best	3.393 × 10^3^	3.643 × 10^3^	3.758 × 10^3^	4.436 × 10^3^	4.251 × 10^3^	4.427 × 10^3^	3.709 × 10^3^	3.655 × 10^3^	4.940 × 10^3^
	Rank	1	5	4	7	6	8	3	2	9
F30	Ave	**2.703 × 10^4^**	5.087 × 10^4^	8.922 × 10^6^	1.603 × 10^8^	2.463 × 10^7^	3.301 × 10^7^	1.178 × 10^6^	6.930 × 10^6^	1.191 × 10^9^
	Std	1.575 × 10^4^	6.900 × 10^4^	9.825 × 10^6^	8.697 × 10^7^	1.589 × 10^7^	2.176 × 10^7^	2.038 × 10^6^	5.327 × 10^6^	5.720 × 10^7^
	Best	8.203 × 10^3^	1.294 × 10^4^	1.040 × 10^6^	5.523 × 10^7^	5.505 × 10^6^	5.552 × 10^6^	1.917× 10^4^	2.344 × 10^6^	2.332 × 10^7^
	Rank	1	2	5	8	6	7	3	4	9
Mean Rank		1.2069	2.4483	5.2759	6.5172	7.3793	6.3448	4.5862	2.6897	8.5517
Result		1	2	5	7	8	6	4	3	9

The bold data represent the optimal average data among all the comparison algorithms.

**Table 4:** Wilcoxon rank sum test values of each comparison algorithm (30-dimensional CEC2017 test set).

Result	Algorithms							
	HBA	SHO	SCA	TSA	WOA	MFO	GWO	AOA
F1	**4.09356 × 10^−^** **^1^**	-	-	-	-	-	-	-
F3	2.30247 × 10^−5^	7.89803 × 10^−8^	-	2.06160 × 10^−6^	-	7.89803 × 10^−8^	1.10447 × 10^−5^	1.65708 × 10^−7^
F4	**3.50702 × 10** **^−^** **^1^**	-	-	-	7.89803 × 10^−8^	7.89803 × 10^−8^	7.94795 × 10^−7^	-
F5	**9.09074 × 10** **^−^** **^2^**	-	-	-	-	7.89803 × 10^−8^	**8.18149 × 10^−^** **^1^**	-
F6	-	-	-	-	-	-	-	-
F7	5.62904 × 10^−4^	-	-	-	-	4.16576 × 10^−5^	1.95335 × 10^−3^	-
F8	**4.56951 × 10** **^−^** **^1^**	1.06457 × 10^−7^	-	-	-	-	**9.67635 × 10^−^** **^1^**	-
F9	2.95975 × 10^−7^	-	-	-	-	-	1.80745 × 10^−5^	-
F10	**5.79218 × 10** **^−^** **^1^**	4.15502 × 10^−4^	-	-	-	3.63883 × 10^−3^	5.56046 × 10^−3^	-
F11	1.59972 × 10^−5^	-	-	-	-	-	-	-
F12	**4.72676 × 10** **^−^** **^1^**	1.82672 × 10^−4^	1.82672 × 10^−4^	1.82672 × 10^−4^	1.82672 × 10^−4^	1.82672 × 10^−4^	1.82672 × 10^−4^	1.82672 × 10^−4^
F13	5.11526 × 10^−3^	-	-	-	7.89803 × 10^−8^	1.20089 × 10^−6^	6.91658 × 10^−7^	-
F14	1.13297 × 10^−2^	1.82672 × 10^−4^	1.82672 × 10^−4^	4.39639 × 10^−4^	1.70625 × 10^−3^	3.76353 × 10^−2^	2.11339 × 10^−2^	1.82672 × 10^−4^
F15	3.60483 × 10^−2^	1.44383 × 10^−4^	-	-	-	1.44383 × 10^−4^	1.20089 × 10^−6^	-
F16	1.79364 × 10^−4^	1.20089 × 10^−6^	-	9.17277 × 10^−8^	-	1.65708 × 10^−7^	3.05663 × 10^−3^	-
F17	2.22203 × 10^−4^	1.57567 × 10^−6^	-	1.20089 × 10^−6^	-	1.04727 × 10^−6^	6.01106 × 10^−2^	-
F18	**9.69850 × 10** **^−^** **^1^**	1.82672 × 10^−4^	1.82672 × 10^−4^	5.82840 × 10^−4^	1.82672 × 10^−4^	1.31494E−03	5.79536 × 10^−3^	1.82672 × 10^−4^
F19	**2.73285 × 10** **^−^** **^1^**	2.56295 × 10^−7^	-	-	-	1.91771 × 10^−7^	1.65708 × 10^−7^	-
F20	2.04071 × 10^−5^	4.53897 × 10^−7^	-	9.17277 × 10^−8^	9.17277 × 10^−8^	2.95975 × 10^−7^	2.22203 × 10^−4^	-
F21	**6.55361 × 10** **^−^** **^1^**	-	-	-	-	2.06160 × 10^−6^	**6.35945 × 10^−^** **^1^**	-
F22	3.49946 × 10^−6^	1.65708 × 10^−7^	7.89803 × 10^−8^	9.17277 × 10^−8^	9.17277 × 10^−8^	1.91771 × 10^−7^	7.94795 × 10^−7^	-
F23	8.35717 × 10^−4^	-	-	-	-	5.22689 × 10^−7^	1.78238 × 10^−3^	-
F24	9.78649 × 10^−3^	-	-	-	-	9.04540 × 10^−3^	8.35717 × 10^−4^	-
F25	**3.23482 × 10** **^−^** **^1^**	-	-	-	-	3.49946 × 10^−6^	7.89803 × 10^−8^	-
F26	**1.19856 × 10** **^−^** **^1^**	-	-	1.20089 × 10^−6^	-	9.17277 × 10^−8^	**1.55570 × 10^−^** **^1^**	-
F27	1.29405 × 10^−4^	-	-	-	7.89803 × 10^−8^	4.68040 × 10^−5^	4.32018 × 10^−3^	**2.85305 × 10^−^** **^1^**
F28	**9.09074 × 10^−^** **^2^**	-	-	-	1.23464 × 10^−7^	1.43085 × 10^−7^	3.41558 × 10^−7^	1.91771 × 10^−7^
F29	1.80297 × 10^−6^	2.21776 × 10^−7^	-	-	-	2.56295 × 10^−7^	4.68040 × 10^−5^	-
F30	**3.23482 × 10** **^−^** **^1^**	-	-	-	-	6.67365 × 10^−6^	-	-
**+/=/−**	3/13/13	0/12/17	0/0/29	0/0/29	0/0/29	0/0/29	1/4/24	0/1/28

The bold data represent *p* values with a significance level greater than 0.05.

**Table 5:** Comparison results of EHBA and other methods on CEC 2020 test sets.

F	Index	Algorithms								
		EHBA	HBA	SHO	SCA	TSA	WOA	MFO	GWO	AOA
F1	Ave	**5.146 × 10^3^**	9.416 × 10^3^	2.123 × 10^10^	1.776 × 10^10^	2.198 × 10^10^	1.568 × 10^9^	8.572 × 10^9^	2.396 × 10^9^	4.962 × 10^10^
	Std	5.482 × 10^3^	1.051 × 10^4^	7.070 × 10^9^	2.384 × 10^9^	1.096 × 10^10^	7.708 × 10^8^	5.976 × 10^9^	2.097 × 10^9^	7.739 × 10^9^
	Best	1.275 × 10^2^	3.130 × 10^2^	5.350 × 10^9^	1.228 × 10^10^	1.057 × 10^10^	8.104 × 10^8^	2.115 × 10^4^	3.908 × 10^7^	3.301 × 10^10^
	Rank	1	2	7	6	8	3	5	4	9
F2	Ave	5.005 × 10^3^	5.007 × 10^3^	5.549 × 10^3^	8.775 × 10^3^	7.437 × 10^3^	7.134 × 10^3^	5.347 × 10^3^	**4.694 × 10^3^**	8.418 × 10^3^
	Std	3.957 × 10^2^	7.068 × 10^2^	4.511 × 10^2^	2.669 × 10^2^	4.619 × 10^2^	1.104 × 10^3^	3.973 × 10^2^	1.321 × 10^3^	5.213 × 10^2^
	Best	4.235 × 10^3^	4.055 × 10^3^	4.440 × 10^3^	8.167 × 10^3^	6.495 × 10^3^	5.392 × 10^3^	4.772 × 10^3^	3.285 × 10^3^	7.589 × 10^3^
	Rank	2	3	5	9	7	6	4	1	8
F3	Ave	**8.701 × 10^2^**	9.070 × 10^2^	1.121 × 10^3^	1.213 × 10^3^	1.210 × 10^3^	1.293 × 10^3^	1.148 × 10^3^	9.049 × 10^2^	1.375 × 10^3^
	Std	2.882 × 10^1^	6.115 × 10^1^	6.218 × 10^1^	6.894 × 10^1^	9.149 × 10^1^	6.968 × 10^1^	1.793 × 10^2^	5.734 × 10^1^	6.906 × 10^1^
	Best	8.255 × 10^2^	8.244 × 10^2^	1.026 × 10^3^	1.117 × 10^3^	1.018 × 10^3^	1.187 × 10^3^	8.803 × 10^2^	8.309 × 10^2^	1.232 × 10^3^
	Rank	1	3	4	7	6	8	5	2	9
F4	Ave	**1.900 × 10^3^**	**1.900 × 10^3^**	**1.900 × 10^3^**	1.912 × 10^3^	1.919 × 10^3^	1.900 × 10^3^	4.454 × 10^4^	**1.900 × 10^3^**	**1.900 × 10^3^**
	Std	0.000 × 10^0^	0.000 × 10^0^	0.000 × 10^0^	8.133 × 10^0^	5.474 × 10^3^	0.000 × 10^0^	5.325 × 10^4^	2.049E−01	0.000 × 10^0^
	Best	1.900 × 10^3^	1.900 × 10^3^	1.900 × 10^3^	1.900 × 10^3^	1.908 × 10^3^	1.900 × 10^3^	1.907 × 10^3^	1.900 × 10^3^	1.900 × 10^3^
	Rank	1	1	1	7	8	1	9	1	1
F5	Ave	2.524 × 10^6^	**3.533 × 10^5^**	9.756 × 10^6^	1.139 × 10^7^	1.433 × 10^7^	1.085 × 10^7^	5.594 × 10^6^	2.112 × 10^6^	8.184 × 10^7^
	Std	1.985 × 10^6^	2.935 × 10^5^	8.155 × 10^6^	3.445 × 10^6^	1.906 × 10^7^	7.497 × 10^6^	6.985 × 10^6^	3.032 × 10^6^	3.949 × 10^7^
	Best	1.437 × 10^5^	4.945 × 10^4^	3.006 × 10^6^	5.768 × 10^6^	2.692 × 10^5^	1.752 × 10^6^	2.402 × 10^5^	1.549 × 10^5^	2.854 × 10^7^
	Rank	3	1	5	7	8	6	4	2	9
F6	Ave	**1.966 × 10^3^**	2.285 × 10^3^	2.295 × 10^3^	3.832 × 10^3^	3.073 × 10^3^	3.638 × 10^3^	2.606 × 10^3^	2.083 × 10^3^	4.109 × 10^3^
	Std	1.016 × 10^2^	3.105 × 10^2^	2.523 × 10^2^	2.371 × 10^2^	7.328 × 10^2^	6.569 × 10^2^	4.053 × 10^2^	1.803 × 10^2^	7.046 × 10^2^
	Best	1.752 × 10^3^	1.745 × 10^3^	1.957 × 10^3^	3.480 × 10^3^	2.006 × 10^3^	2.529 × 10^3^	1.909 × 10^3^	1.783 × 10^3^	2.997 × 10^3^
	Rank	1	3	4	8	6	7	5	2	9
F7	Ave	**4.674 × 10^5^**	1.039 × 10^6^	1.575 × 10^6^	4.186 × 10^6^	3.523 × 10^6^	1.018 × 10^7^	1.409 × 10^6^	2.254 × 10^6^	2.817 × 10^7^
	Std	3.712 × 10^5^	4.159 × 10^6^	3.022 × 10^6^	3.272 × 10^6^	4.506 × 10^6^	8.030 × 10^6^	1.402 × 10^6^	3.431 × 10^6^	1.977 × 10^7^
	Best	1.085 × 10^5^	1.666 × 10^4^	9.209 × 10^4^	6.239 × 10^5^	8.674 × 10^4^	7.705 × 10^5^	8.947 × 10^4^	1.207 × 10^5^	5.958 × 10^6^
	Rank	1	2	4	7	6	8	3	5	9
F8	Ave	**2.760 × 10^3^**	4.184 × 10^3^	6.149 × 10^3^	9.928 × 10^3^	8.306 × 10^3^	7.083 × 10^3^	6.621 × 10^3^	5.419 × 10^3^	9.160 × 10^3^
	Std	1.418 × 10^3^	2.438 × 10^3^	1.242 × 10^3^	1.196 × 10^3^	1.604 × 10^3^	1.933 × 10^3^	1.109 × 10^3^	2.042 × 10^3^	1.592 × 10^3^
	Best	2.300 × 10^3^	2.300 × 10^3^	3.994 × 10^3^	5.288 × 10^3^	3.975 × 10^3^	2.793 × 10^3^	3.695 × 10^3^	2.461 × 10^3^	5.740 × 10^3^
	Rank	1	2	4	9	7	6	5	3	8
F9	Ave	2.952 × 10^3^	3.015 × 10^3^	3.283 × 10^3^	3.228 × 10^3^	3.396 × 10^3^	3.263 × 10^3^	2.999 × 10^3^	**2.942 × 10^3^**	3.864 × 10^3^
	Std	3.252 × 10^1^	1.594 × 10^2^	6.334 × 10^1^	3.797 × 10^1^	1.185 × 10^2^	9.144 × 10^1^	3.562 × 10^1^	6.694 × 10^1^	1.991 × 10^2^
	Best	2.914 × 10^3^	2.894 × 10^3^	3.165 × 10^3^	3.155 × 10^3^	3.228 × 10^3^	3.073 × 10^3^	2.925 × 10^3^	2.867 × 10^3^	3.431 × 10^3^
	Rank	2	4	7	5	8	6	3	1	9
F10	Ave	**2.899 × 10^3^**	2.904 × 10^3^	3.520 × 10^3^	3.451 × 10^3^	3.602 × 10^3^	3.135 × 10^3^	3.398 × 10^3^	3.013 × 10^3^	4.774 × 10^3^
	Std	1.665 × 10^1^	1.847 × 10^1^	2.674 × 10^2^	1.503 × 10^2^	4.233 × 10^2^	4.906 × 10^1^	4.504 × 10^2^	6.055 × 10^1^	5.229 × 10^2^
	Best	2.884 × 10^3^	2.884 × 10^3^	3.117 × 10^3^	3.186 × 10^3^	3.086 × 10^3^	3.054 × 10^3^	2.896 × 10^3^	2.933 × 10^3^	3.875 × 10^3^
	Rank	1	2	7	6	8	4	5	3	9
Mean Rank		1.4	2.3	4.8	7.1	7.2	5.5	4.8	2.4	8
Result		1	2	4	6	7	6	4	3	9

The bold data represent the optimal average data among all the comparison algorithms.

**Table 6:** Wilcoxon rank sum test values of each comparison algorithm on CEC2020 test set.

Result	Algorithms							
	HBA	SHO	SCA	TSA	WOA	MFO	GWO	AOA
F1	4.1124 × 10^−2^	-	-	-	-	-	-	-
F2	**6.5536 × 10** **^−^** **^1^**	4.1550 × 10^−4^	-	-	2.2178 × 10^−7^	2.9441 × 10^−2^	1.4810 × 10^−3^	-
F3	**7.2045 × 10** **^−^** **^2^**	-	-	-	-	1.9177 × 10^−7^	2.3903 × 10^−2^	-
F4	NaN	NaN	8.0065 × 10^−9^	8.0065 × 10^−9^	NaN	8.0065 × 10^−9^	3.2162 × 10^−6^	NaN
F5	5.8736 × 10^−6^	1.4149 × 10^−5^	1.0646 × 10^−7^	4.3202 × 10^−3^	1.1045 × 10^−5^	**1.4042 × 10^−^** **^1^**	**1.1986 × 10^−^** **^1^**	-
F6	9.2091 × 10^−4^	7.5774 × 10^−6^	-	1.6571 × 10^−7^	-	1.0473 × 10^−6^	3.1517 × 10^−2^	-
F7	8.2924 × 10^−5^	**6.7868 × 10^−2^**	2.9598 × 10^−7^	3.0566 × 10^−3^	1.2346 × 10^−7^	1.1433 × 10^−2^	**5.0751 × 10** **^−^** **^1^**	-
F8	3.7499 × 10^−4^	2.6898 × 10^−6^	9.1728 × 10^−8^	1.2346 × 10^−7^	3.9388 × 10^−7^	1.5757 × 10^−6^	5.8736 × 10^−6^	1.6571 × 10^−7^
F9	**2.2869 × 10^−^** **^1^**	-	-	-	-	6.6104 × 10^−5^	**6.3892 × 10** **^−^** **^2^**	-
F10	**3.6484 × 10^−^** **^1^**	-	-	-	-	3.4156 × 10^−7^	1.0646 × 10^−7^	-
**+/=/−**	1/5/4	0/2/8	0/0/10	0/0/10	0/1/9	0/1/9	3/3/4	0/1/9

The bold data represent *p* values with a significance level greater than 0.05.

**Table 7:** Comparison results of EHBA and other methods on CEC2022 test set.

F	Index	Algorithms								
		EHBA	HBA	SHO	SCA	TSA	WOA	MFO	GWO	AOA
F1	Ave	**5.364 × 10^3^**	1.104 × 10^4^	1.617 × 10^4^	1.497 × 10^4^	1.579 × 10^4^	2.766 × 10^4^	3.116 × 10^4^	1.430 × 10^4^	1.954 × 10^4^
	Std	2.919 × 10^3^	4.507 × 10^4^	4.914 × 10^3^	3.327 × 10^3^	5.756 × 10^3^	9.329 × 10^3^	2.064 × 10^4^	4.016 × 10^3^	3.527 × 10^3^
	Best	1.098 × 10^3^	3.200 × 10^3^	9.519 × 10^3^	8.203 × 10^3^	9.326 × 10^3^	1.648 × 10^4^	5.139 × 10^3^	6.364 × 10^3^	1.039 × 10^4^
	Rank	1	2	6	4	5	8	9	3	7
F2	Ave	**4.511 × 10^2^**	4.589 × 10^2^	7.055 × 10^2^	7.453 × 10^2^	7.319 × 10^2^	5.662 × 10^2^	5.508 × 10^2^	5.013 × 10^2^	1.945 × 10^3^
	Std	8.416 × 10^0^	1.438 × 10^1^	1.190 × 10^2^	8.521 × 10^1^	1.745 × 10^2^	5.987 × 10^1^	1.236 × 10^2^	4.262 × 10^1^	5.160 × 10^2^
	Best	4.449 × 10^2^	4.290 × 10^2^	5.753 × 10^2^	6.206 × 10^2^	4.925 × 10^1^	4.594 × 10^2^	4.449 × 10^2^	4.540 × 10^2^	1.290 × 10^3^
	Rank	1	2	6	8	7	5	4	3	9
F3	Ave	**6.001 × 10^2^**	6.053 × 10^2^	6.383 × 10^2^	6.462 × 10^2^	6.630 × 10^2^	6.666 × 10^2^	6.221 × 10^2^	6.055 × 10^2^	6.646 × 10^2^
	Std	3.968 × 10^−1^	3.002 × 10^0^	5.963 × 10^0^	4.799 × 10^0^	1.587 × 10^1^	1.215 × 10^1^	1.055 × 10^1^	3.531 × 10^0^	6.684 × 10^0^
	Best	6.000 × 10^2^	6.013 × 10^2^	6.247 × 10^2^	6.367 × 10^2^	6.289 × 10^2^	6.340 × 10^2^	6.092 × 10^2^	6.008 × 10^2^	6.528 × 10^2^
	Rank	1	2	5	6	7	9	4	3	8
F4	Ave	8.537 × 10^2^	8.541 × 10^2^	8.965 × 10^2^	9.462 × 10^2^	9.548 × 10^2^	9.337 × 10^2^	8.908 × 10^2^	**8.535 × 10^2^**	9.408 × 10^2^
	Std	1.346 × 10^1^	1.574 × 10^1^	1.796 × 10^1^	1.282 × 10^1^	2.473 × 10^1^	3.202 × 10^1^	2.209 × 10^1^	2.206 × 10^1^	1.269 × 10^1^
	Best	8.301 × 10^2^	8.239 × 10^2^	8.707 × 10^2^	9.193 × 10^2^	9.157 × 10^2^	8.791 × 10^2^	8.413 × 10^2^	8.231 × 10^2^	9.141 × 10^2^
	Rank	2	3	5	8	9	6	4	1	7
F5	Ave	**1.053 × 10^3^**	1.401 × 10^3^	2.403 × 10^3^	2.488 × 10^3^	4.692 × 10^3^	3.664 × 10^3^	2.987 × 10^3^	1.193 × 10^3^	2.843 × 10^3^
	Std	2.894 × 10^2^	3.605 × 10^2^	1.908 × 10^2^	4.189 × 10^2^	1.698 × 10^3^	1.372 × 10^3^	1.105 × 10^3^	2.092 × 10^2^	4.380 × 10^2^
	Best	9.008 × 10^2^	9.116 × 10^2^	2.047 × 10^3^	1.663 × 10^3^	2.126 × 10^3^	1.901 × 10^3^	1.241 × 10^3^	9.158 × 10^2^	2.082 × 10^3^
	Rank	1	3	4	5	9	8	7	2	6
F6	Ave	**7.741 × 10^3^**	9.352 × 10^3^	4.906 × 10^6^	9.297 × 10^7^	2.896 × 10^8^	1.105 × 10^6^	1.035 × 10^8^	7.845 × 10^6^	1.024 × 10^9^
	Std	6.441 × 10^3^	8.701 × 10^3^	9.685 × 10^6^	6.017 × 10^6^	7.746 × 10^8^	1.472 × 10^6^	4.192 × 10^8^	1.549 × 10^7^	6.891 × 10^8^
	Best	2.355 × 10^3^	1.957 × 10^3^	1.695 × 10^4^	1.740 × 10^7^	3.064 × 10^5^	2.296 × 10^4^	2.521 × 10^3^	2.558 × 10^3^	9.256 × 10^7^
	Rank	1	2	4	6	8	3	7	5	9
F7	Ave	**2.046 × 10^3^**	2.062 × 10^3^	2.120 × 10^3^	2.149 × 10^3^	2.267 × 10^3^	2.230 × 10^3^	2.123 × 10^3^	2.074 × 10^3^	2.174 × 10^3^
	Std	3.150 × 10^1^	1.698 × 10^1^	2.485 × 10^1^	2.150 × 10^1^	1.252 × 10^2^	7.125 × 10^1^	5.616 × 10^1^	3.165 × 10^1^	2.548 × 10^1^
	Best	2.024 × 10^3^	2.039 × 10^3^	2.065 × 10^3^	2.110 × 10^3^	2.127 × 10^3^	2.098 × 10^3^	2.03 × 10^3^	2.034 × 10^3^	2.128 × 10^3^
	Rank	1	2	4	6	9	8	5	3	7
F8	Ave	**2.224 × 10^3^**	2.273 × 10^3^	2.261 × 10^3^	2.275 × 10^3^	2.409 × 10^3^	2.289 × 10^3^	2.264 × 10^3^	2.267 × 10^3^	2.275 × 10^3^
	Std	1.569 × 10^0^	6.082 × 10^1^	4.657 × 10^1^	2.134 × 10^1^	4.029 × 10^2^	6.970 × 10^1^	4.653 × 10^1^	5.435 × 10^1^	8.732 × 10^1^
	Best	2.223 × 10^3^	2.223 × 10^3^	2.227 × 10^3^	2.243 × 10^3^	2.236 × 10^3^	2.232 × 10^3^	2.223 × 10^3^	2.226 × 10^3^	2.232 × 10^3^
	Rank	1	5	2	6	9	8	3	4	7
F9	Ave	**2.481 × 10^3^**	2.481 × 10^3^	2.595 × 10^3^	2.599 × 10^3^	2.674 × 10^3^	2.579 × 10^3^	2.513 × 10^3^	2.522 × 10^3^	3.173 × 10^3^
	Std	2.371 × 10^−4^	5.208 × 10^−2^	4.287 × 10^1^	2.776 × 10^1^	8.627 × 10^1^	4.222 × 10^1^	3.371 × 10^1^	3.303 × 10^1^	2.213 × 10^2^
	Best	2.481 × 10^3^	2.481 × 10^3^	2.514 × 10^3^	2.551 × 10^3^	2.580 × 10^3^	2.524 × 10^3^	2.481 × 10^3^	2.481 × 10^3^	2.826 × 10^3^
	Rank	1	2	6	7	8	5	3	4	9
F10	Ave	**2.471 × 10^3^**	3.731 × 10^3^	3.202 × 10^3^	3.104 × 10^3^	5.402 × 10^3^	4.747 × 10^3^	3.970 × 10^3^	3.323 × 10^3^	4.838 × 10^3^
	Std	4.709 × 10^1^	1.164 × 10^3^	6.864 × 10^2^	1.293 × 10^3^	8.277 × 10^2^	1.097 × 10^3^	1.087 × 10^3^	8.470 × 10^2^	1.671 × 10^3^
	Best	2.404 × 10^3^	2.501 × 10^3^	2.521 × 10^3^	2.526 × 10^3^	2.809 × 10^3^	2.501 × 10^3^	2.501 × 10^3^	2.500 × 10^3^	2.624 × 10^3^
	Rank	1	5	3	2	9	7	6	4	8
F11	Ave	**2.900 × 10^3^**	2.900 × 10^3^	5.207 × 10^3^	4.542 × 10^3^	5.785 × 10^3^	3.788 × 10^3^	3.756 × 10^3^	3.430 × 10^3^	7.983 × 10^3^
	Std	1.124 × 10^2^	7.947 × 10^1^	5.532 × 10^2^	5.781 × 10^2^	1.228 × 10^3^	9.650 × 10^2^	6.090 × 10^2^	2.009 × 10^2^	5.790 × 10^2^
	Best	2.600 × 10^3^	2.600 × 10^3^	4.054 × 10^3^	3.742 × 10^3^	3.961 × 10^3^	2.866 × 10^3^	2.900 × 10^3^	3.131 × 10^3^	7.016 × 10^3^
	Rank	2	1	7	6	8	5	4	3	9
F12	Ave	2.970 × 10^3^	3.091 × 10^3^	3.174 × 10^3^	3.072 × 10^3^	3.301 × 10^3^	3.042 × 10^3^	**2.960 × 10^3^**	2.972 × 10^3^	3.455 × 10^3^
	Std	4.170 × 10^1^	1.030 × 10^2^	9.535 × 10^1^	3.083 × 10^1^	1.789 × 10^2^	6.701 × 10^1^	1.533 × 10^1^	2.737 × 10^1^	4.983 × 10^2^
	Best	2.944 × 10^3^	2.960 × 10^3^	3.059 × 10^3^	3.026 × 10^3^	2.987 × 10^3^	2.960 × 10^3^	2.942 × 10^3^	2.946 × 10^3^	2.900 × 10^3^
	Rank	2	6	7	5	8	4	1	3	9
Mean Rank		1.2500	2.9167	4.9167	5.7500	8.0000	6.3333	4.7500	3.1667	7.9167
Result		1	2	5	6	8	7	4	3	9

The bold data represent the optimal average data among all the comparison algorithms.

**Table 8:** Wilcoxon rank sum test values of each comparison algorithm on CEC2022 test set.

Result	Algorithms							
	HBA	SHO	SCA	TSA	WOA	MFO	GWO	AOA
F1	7.5774 × 10^−6^	1.2346 × 10^−7^	1.6571 × 10^−7^	1.9177 × 10^−7^	-	1.8030 × 10^−6^	5.2269 × 10^−7^	7.8980 × 10^−8^
F2	**4.0936 × 10^−1^**	-	-	-	9.1728 × 10^−8^	1.2941 × 10^−4^	3.4156 × 10^−7^	-
F3	9.1728 × 10^−8^	-	-	-	-	-	1.0646 × 10^−7^	-
F4	**8.6043 × 10^−1^**	1.2346 × 10^−7^	-	-	-	3.9874 × 10^−6^	**6.1677 × 10** **^−^** **^1^**	-
F5	2.4706 × 10^4^	1.0646 × 10^−7^	1.2346 × 10^−7^	7.8980 × 10^−8^	7.8980 × 10^−8^	1.9177 × 10^−7^	1.9533 × 10^3^	7.8980 × 10^−8^
F6	**8.3923 × 10^−1^**	1.6571 × 10^−7^	-	-	7.8980 × 10^−8^	9.7865 × 10^−3^	7.4064 × 10^−5^	-
F7	9.2780 × 10^−5^	1.2009 × 10^−6^	6.9166 × 10^−7^	1.9177 × 10^−7^	1.4309 × 10^−7^	1.1045 × 10^−5^	4.1658 × 10^−5^	3.9388 × 10^−7^
F8	7.4064 × 10^−5^	1.0646 × 10^−7^	-	-	-	1.8030 × 10^−6^	6.0148 × 10^−7^	-
F9	2.4706 × 10^−4^	-	-	-	-	-	-	-
F10	1.2505 × 10^−5^	9.1728 × 10^−8^	2.5629 × 10^−7^	-	2.5629 × 10^−7^	2.5629 × 10^−7^	1.1590 × 10^−4^	-
F11	**7.6431 × 10^−2^**	-	-	-	9.1266 × 10^−7^	1.5997 × 10^−5^	-	-
F12	3.0691 × 10^−6^	2.2178 × 10^−7^	1.2009 × 10^−6^	2.2178 × 10^−7^	8.5974 × 10^−6^	4.9033 × 10^−1^	2.1841 × 10^−1^	1.1355 × 10^−1^
**+/=/−**	1/4/7	0/0/12	0/0/12	0/0/12	0/0/12	1/1/10	0/2/10	0/1/11

The bold data represent *p* values with a significance level greater than 0.05.

**Table 9:** Statistical results of the welded beam problem.

Methods	Optimum	Mean	Worst	Std
EHBA	**1.4337819**	1.4349374	1.4364151	0.0007941
HBA	1.4338074	1.4338090	1.4338362	0.0000064
SHO	1.4446079	1.5038656	1.5661039	0.0349623
SCA	1.4831125	1.5398212	1.5972414	0.0277449
TSA	1.4410437	1.4476576	1.4557406	0.0037096
WOA	1.4666907	1.9728059	3.4551200	0.5119476
MFO	1.4338074	1.4907124	1.8869767	0.1384304
GWO	1.4345662	1.4379413	1.4472849	0.0036150
AOA	1.6202084	1.9650833	2.3842840	0.2300621

**Table 10:** Optimal results of the welded beam problem.

Methods	Variables				Optimum
	*γ*_1_	*γ*_2_	*γ*_3_	*γ*_4_	
EHBA	0.2053846	1.3360529	9.0363311	0.2057430	1.4337819
HBA	0.2057298	1.3335605	9.0366239	0.2057296	1.4338074
SHO	0.1897981	1.4872939	9.0352177	0.2057937	1.4446079
SCA	0.1764307	1.5879257	9.1993613	0.2070625	1.4831125
TSA	0.2016789	1.3854980	9.0578041	0.2056496	1.4410437
WOA	0.1711888	1.7247850	9.0084039	0.2070206	1.4666907
MFO	0.2057298	1.3335604	9.0366239	0.2057296	1.4338074
GWO	0.2054924	1.3395471	9.0364999	0.2057456	1.4345662
AOA	0.1810423	1.5299606	10.0000000	0.2094384	1.6202084

**Table 11:** Optimal results of side impact design problems for cars.

Methods	Variables											Optimum
	*γ*_1_	*γ*_2_	*γ*_3_	*γ*_4_	*γ*_5_	*γ*_6_	*γ*_7_	*γ*_8_	*γ*_9_	*γ*_10_	*γ*_11_	
EHBA	0.5000	1.0525	0.5000	0.5000	0.5000	1.5000	0.5000	0.3450	0.3450	−30.0000	0.0000	**22.2383119**
HBA	0.5000	1.0525	0.5000	0.5000	0.5000	1.5000	0.5000	0.3450	0.3450	−30.0000	0.0000	**22.2383119**
SHO	0.5167	1.0452	0.5000	0.5000	0.5000	1.4948	0.5000	0.3437	0.3309	−29.9994	−0.0346	22.2725325
SCA	0.5000	1.0589	0.5000	0.5000	0.5000	1.4546	0.5000	0.3450	0.3450	−30.0000	−0.1623	22.3236875
TSA	0.5000	1.0532	0.5000	0.5000	0.5000	1.5000	0.5000	0.3450	0.3450	−30.0000	−1.0450	22.2398005
WOA	0.5000	1.0525	0.5000	0.5000	0.5000	1.5000	0.5000	0.3450	0.3450	−30.0000	6.4878	22.2383130
MFO	0.5000	1.0525	0.5000	0.5000	0.5000	1.5000	0.5000	0.3450	0.3450	−30.0000	0.0000	**22.2383119**
GWO	0.5315	1.0386	0.5000	0.5000	0.5000	1.5000	0.5000	0.3450	0.3450	−29.9964	0.0384	22.2390089
AOA	0.5000	1.0964	0.5000	0.5000	0.5000	1.5000	0.5000	0.3450	0.1920	−26.8686	−0.0529	22.4954648

**Table 12:** Statistical results of vehicle side impact design issues.

Methods	Optimum	Mean	Worst	Std
EHBA	22.2383119	22.2383145	22.2383439	0.0000075
HBA	22.2383119	22.7318745	25.1456712	0.7577413
SHO	22.2725325	22.4135001	22.5908204	0.0807070
SCA	22.3236875	22.8925219	23.5196336	0.3130891
TSA	22.2398005	22.4264527	25.4375037	0.7089918
WOA	22.2383130	22.9663887	24.5497539	0.7826121
MFO	22.2383119	22.2832051	22.9923773	0.1680703
GWO	22.2390089	22.2508953	22.2878793	0.0159526
AOA	22.4954648	23.6330412	25.9671223	0.9988731

**Table 13:** Optimal results for parameter estimation of FM sound waves.

Methods	Variables						Optimum
	*γ*_1_	*γ*_2_	*γ*_3_	*γ*_4_	*γ*_5_	*γ*_6_	
EHBA	0.9992900	5.0002498	−1.5010887	4.7998468	2.0002121	4.9000398	**0.0000370**
HBA	0.6268062	−0.0273601	4.3856049	−4.8936163	−0.1260477	−5.1736205	10.9422767
SHO	1.0962431	0.0355469	−0.6068540	−0.0416925	4.2924703	4.8843543	9.6438934
SCA	−0.5006558	−0.0466897	4.4856557	4.8840717	−0.0002421	0.8250744	12.6418622
TSA	0.6205581	0.0240913	4.3344360	−4.7443413	4.0024033	−0.0372704	11.6162542
WOA	0.7647151	0.1268153	−1.1065172	−0.1419302	4.1776909	4.9035396	9.0496958
MFO	0.8563265	4.9215368	−1.1521163	2.4954631	4.9330869	2.4246832	11.2071984
GWO	0.8486533	5.0087885	1.4857537	−4.7910967	1.9845038	−4.9010655	0.7131581
AOA	0.7489891	0.0912872	0.9725311	0.0878281	4.4087630	−4.8947747	9.2791912

**Table 14:** Statistical results of FM sound wave parameter estimation problem.

Methods	Optimum	Mean	Worst	Std
EHBA	0.0000370	9.4855667	20.1670529	6.7437197
HBA	10.9422767	17.6689836	23.1827480	3.4680614
SHO	9.6438934	19.3259034	25.1632318	6.4388645
SCA	12.6418622	21.7408845	24.9466231	2.6678648
TSA	11.6162542	19.4359242	25.2052649	4.2413626
WOA	9.0496958	19.3233360	25.0808811	4.7363808
MFO	11.2071984	19.7214875	27.4896812	6.7926567
GWO	0.7131581	16.0919444	25.0430495	6.4865698
AOA	9.2791912	25.6683935	29.8550028	5.6292789

## Data Availability

All data generated or analyzed during the study are included in this published article.
